# The Draft Genome of Yellow Stem Borer, an Agriculturally Important Pest, Provides Molecular Insights into Its Biology, Development and Specificity Towards Rice for Infestation

**DOI:** 10.3390/insects12060563

**Published:** 2021-06-19

**Authors:** Divya Kattupalli, Kalyani M. Barbadikar, Vishalakshi Balija, Suneel Ballichatla, Athulya R, Ayyagari Phani Padmakumari, Swati Saxena, Kishor Gaikwad, Sridhar Yerram, Premalatha Kokku, Maganti Sheshu Madhav

**Affiliations:** 1 Biotechnology Section, Division of Crop Improvement, ICAR-Indian Institute of Rice Research, Hyderabad 500030, India; divya.kattupalli9@gmail.com (D.K.); kalyaniaau@gmail.com (K.M.B.); vishalakshi.biotech@gmail.com (V.B.); suneel.biotechnology@gmail.com (S.B.); 2 Entomology Section, Division of Crop Protection, ICAR-Indian Institute of Rice Research, Hyderabad 500030, India; athulyakichu@gmail.com (A.R.); padmakumariento@gmail.com (A.P.P.); yerramsridhar303@gmail.com (S.Y.); 3 Genomics Lab, ICAR-National Institute of Plant Biotechnology, New Delhi 110012, India; swatisaxena605@gmail.com (S.S.); kish2012@nrcpb.org (K.G.); 4 Department of Chemistry, Osmania University, Hyderabad 500007, India; premasheshu@gmail.com

**Keywords:** YSB genome, *Scirpophaga*, monophagous, rice pest, effectors, venom-like proteins, insect pest, immune genes

## Abstract

**Simple Summary:**

Yellow stem borer (YSB), is the most destructive and widely occurring pest that attacks rice throughout the growing season. Rice (*Oryza sativa* L.) is a major staple cereal worldwide, providing essential caloric requirements for more than half of the world’s population. Annual losses to rice borers are approximately 5–10%, but losses in individual fields may reach up to 50–60%. The use of traditional pest management strategies in controlling YSB is somewhat challenging due to its unique internal feeding habit. Genome sequence information of economically important crop pests is important for designing or developing pest-resistant rice varieties. In an approach to achieve this, we present our first-ever study on the draft genome sequence of YSB. The information provided from our current study might be useful in developing genome-based approaches for the management of pest species.

**Abstract:**

Yellow stem borer (YSB), *Scirpophaga incertulas* (Walker) (Lepidoptera: Crambidae), a major monophagous insect pest of rice, causes significant yield losses. The rice–YSB interaction is very dynamic, making it difficult for management. The development of resistant lines has been unsuccessful as there are no effective resistant sources in the germplasm. Genome information is necessary for a better understanding of interaction with rice in terms of its recognition, response, and infestation mechanism. The draft genome of YSB is predicted to have 46,057 genes with an estimated size of 308 Mb, being correlated with the flow cytometry analysis. The existence of complex metabolic mechanisms and genes related to specific behavior was identified, being conditioned by a higher level of regulation. We deciphered the possible visual, olfactory, and gustatory mechanisms responsible for its evolution as a monophagous pest. Comparative genomic analysis revealed that YSB is unique in the way it has evolved. The obvious presence of high-immunity-related genes, well-developed RNAi machinery, and diverse effectors provides a means for developing genomic tools for its management. The identified 21,696 SSR markers can be utilized for diversity analysis of populations across the rice-growing regions. We present the first draft genome of YSB. The information emanated paves a way for biologists to design novel pest management strategies as well as for the industry to design new classes of safer and specific insecticide molecules.

## 1. Introduction

A large section of the world population is perpetually fighting hunger and malnutrition. This demands an increase in the yields of major food crops such as rice, wheat, and maize [1,2]. Global climate change, depleting resources, and increasing use of chemicals for control of disease/pests also pose a bigger challenge for food and nutritional security. Rice (*Oryza sativa*) is the most widely grown crop in the Asian continent as about 90% of rice production and consumption takes place in this region [3,4]. The two major factors that are responsible for considerable yield loss in rice are regular pest outbreaks and adverse weather conditions. World rice production is being affected by several insect pests that cause an average yield loss of up to 20% in Asia, with the damage being largely done by stem borers, leafhoppers and planthoppers, gall midge, and grain-sucking bugs [5]. Among them, the yellow stem borer (YSB), *Scirpophaga incertulas* (Walker) (Lepidoptera), is the most destructive widely occurring pest that attacks rice throughout the growing season [6,7]. Newly emerged larvae enter the stem for feeding on the internal tissues at vegetative and reproductive stages of the plant growth, leading to the formation of ‘dead hearts’ and ‘white ears’. Annual losses due to rice borers are approximately 5–10%, but losses in individual fields may reach 50–60% [8,9,10]. Recovery or prevention of 5% of the losses to stem borers could feed approximately 140 million people for one year [10]. Due to its internal feeding habit, management of the pest becomes a challenge with conventional methods of pest management. Genome sequence information of economically important crop pests is important to provide genetic tools for designing next-generation pest-resistant rice [11]. The development of such genomic data is crucial for understanding the epidemiological, evolutionary, and behavioral characteristics of crop pests. The importance of genomic information is evident with the increasing number of insect genomes being reported [12].

However, genomic resources are sparsely available for YSB. With this background, genome information of YSB can provide insights into the structure, function, biology, molecular mechanisms, monophagy, and gene regulation. We sequenced the YSB genome using Illumina short reads and mate-pair technology and assembled the sequences de novo. The data emanated from this work can aid in exploring genome-based approaches for the management of YSB.

## 2. Materials and Methods

### 2.1. YSB Sampling and Sequencing

Adults of YSB were collected from one plot of ICAR-IIRR, Hyderabad farm, and females were selected based on the morphological features and were subjected to whole-genome sequencing [13,14]. The DNA was isolated from the female adult YSB using a standard CTAB method and checked for quality and quantity on 0.8% agarose gel and Nanodrop, respectively. Two high-throughput sequencing chemistries, namely, Illumina paired-end and Nextera Mate pair, were employed for sequencing the DNA. The mate-pair sequencing library was prepared using Illumina Nextera Mate Pair sample preparation kit and the paired-end sequencing library was prepared using Illumina TruSeq standard Nano DNA Library Prep Kit using the manufacturer’s protocols. All the steps were followed accordingly and verified as per the standard manufacturer’s protocol. The PCR amplified libraries were analyzed on Tape Station 4200 (Agilent Technologies, Santa Clara, CA, USA) using High-Sensitivity D1000 Screen Tape assay kit as per the manufacturer instructions. The libraries (mean library fragments of 621 bp for Mate-Pair and 415 bp for Shotgun library) were loaded onto NexSeq 500 (2 × 150 bp) for cluster generation and sequencing.

### 2.2. De Novo Assembly and Gene Prediction 

The sequenced data were processed for quality filtering using Trimmomatic v0.35 to remove adapter sequences, ambiguous reads (reads with unknown nucleotides “N” larger than 5%), and low-quality sequences (read with more than 10% quality threshold (QV) < 20 Phred score). The filtered high-quality paired-end (PE) and mate-pair (MP) reads of the YSB sample were assembled into scaffolds using SPAdes (v.3.7.1, http://bioinf.spbau.ru/spades, accessed on 15 January 2020) assembler followed by scaffolding using SSPACE. Gene prediction was carried out for the assembled scaffolds of YSB using the AUGUSTUS-3.2.1 gene prediction program. As a model organism, *Tribolium castaneum* was used to predict genes from the assembled YSB scaffolds.

Completeness of genome assembly was checked using CEGMA (Core Eukaryotic Genes Mapping Approach) and BUSCO (Benchmarking Universal Single-Copy Orthologs) available on the gVolante web interface (https://gvolante.riken.jp/faq.html, accessed on 1 August 2020). The YSB scaffolds fasta file was used as input for CEGMA (with core gene set reference from non-vertebrate) and BUSCO (with Arthropoda gene reference set) analyzed with default parameters.

### 2.3. Non-Coding RNA Identification

The 310,612 scaffolds were subjected to transfer RNA (tRNA) and ribosomal RNA (rRNA) prediction. The tRNAs were predicted using tRNA scan-SE (http://lowelab.ucsc.edu/tRNAscan-SE/, accessed on 15 January 2020). Homology based ribosomal RNAs were predicted using blastN against RNAmmer 1.2 database (http://www.cbs.dtu.dk/services/RNAmmer/, accessed on 1 December 2019) with an e-value cut-off of 1 × 10^−5^.

### 2.4. Repeat Element Identification and SSR Mining 

The assembled scaffolds were subjected to Repeat Masker (version-4.0.7, (http://www.repeatmasker.org/, accessed on 30 January 2020) for the identification of repeat elements in YSB. The simple sequence repeat markers were identified using MIcroSAtellite (MISA) with the following specifications for unit size/minimum number of repeats: 2/6, 3/5, 4/5, 5/5, and 6/5 with 100 bp maximum distance between two SSR repeat motifs [15]. The developed SSR markers were named with the prefix YSB-SSR. The newly developed SSR markers were selected for validation, based on their functional annotation [16]. Based on function, we validated 25 SSR markers in 4 natural YSB populations collected from four locations (Medak (M), Nizamabad (N), Rajendranagar (R), and Warangal (W)) of Telangana state. The genomic DNA was isolated using the CTAB method as described earlier. A touchdown PCR thermal profile was performed for 94 °C for 5 min of initial denaturation, followed by the first five cycles of 94 °C for 30 s, 65 °C to 60 °C for 30 s, and 72 °C for 1 min, with 1 °C decrement in annealing temperature per cycle, then 30 cycles of 94 °C for 30 s with a constant annealing temperature of 60 °C for 30 s and 72 °C for 1 min followed by a final extension for 7 min at 72 °C. Amplified PCR products were separated on 3% metaphor agarose gel (0.5 µg/mL EtBr) along with 50 bp DNA ladder (Fermentas, MA, USA) in 1X TBE buffer at constant power 120 V for about 2.5–3 h. The gels were visualized and documented by a gel documentation system (Bio-Rad, Hercules, CA, USA). The amplified bands were scored as presence (1) or absence (0).

### 2.5. Gene annotation, Gene Ontology, KEGG Pathway, and Cluster of Orthologous Groups (COG) Analysis

Functional annotation of the genes was performed using the BLASTx program, using the NCBI-blast-2.3.0+ standalone tool. BLASTx finds the homologous sequences for the predicted genes against the NR (non-redundant protein) database. Gene ontology (GO) analysis was performed using Blast2GO PRO and provided ontology of defined terms representing gene product properties which are grouped into three main domains, namely, biological process, molecular function, and cellular component. The 46,057 genes were mapped to reference canonical pathways using the KEGG pathway for identification of their potential involvement in the metabolic pathways. The cluster of orthologous groups was identified using eggnog-mapper (http://eggnogdb.embl.de/#/app/emapper, accessed on 20 January 2020) from the predicted protein sequences of YSB.

### 2.6. Phylogenetic Analysis

A representative gene from each functionally significant category as described in the results section was selected from YSB genomic data (having the maximum nucleotide length). These genes were run using NCBI-BLASTx with default parameters (https://blast.ncbi.nlm.nih.gov/, accessed on 30 January 2020). Sequences showing the maximum similarity with other rice pests and non-rice pests were downloaded (until January 2020). A neighbor-joining tree was constructed using MEGA software with 1000 times of bootstrapping. The evolutionary distances were computed using the maximum composite likelihood method [17].

### 2.7. Comparative Genomics

Comparative genomics was carried with the predicted protein sequences of YSB and protein sequences of *C. suppressalis, N. lugens, B. mori,* and *L. striatellus* using Omicsbox bioinformatic tool (https://www.biobam.com/omicsbox/, accessed on 30 January 2020) and Venny tool (https://bioinfogp.cnb.csic.es/tools/venny/, accessed on 31 January 2020). 

### 2.8. Flow Cytometry Using Fluorescence-Activated Cell Sorting (FACS) Method

#### 2.8.1. Plant Material

For this study, we used three standards: *Pisum sativum* (genotype DDR 55 tested with 2C = 9.09 pg) as the primary reference, and *Glycine max* (genotype DS-9712 tested with 2C = 2.5 pg) and *Oryza sativa* (genotype PMK 2 with 2C = 0.90 pg) as a secondary reference; *D. melanogaster* was used as an internal control.

#### 2.8.2. Buffer Preparation

Galbraith’s buffer with a final concentration of 45 mM MgCl_2_, 30 mM sodium citrate, 20 mM MOPS (4-morpholino propane sulfonate), and 0.1% (*v*/*v*) Triton X-100 was used for suspending nuclei from the sample tissues. The pH of the buffer was adjusted to 7.0 using 1N NaOH, and it was filtered through a 0.22 μm filter (Millipore, Merck KGaA, Darmstadt, Germany) and stored at −20 °C. Propidium iodide @ 50 μg/mL (PI; HiMedia, Kennett Square, PA, USA) was freshly prepared on ice just before use. 

#### 2.8.3. Sample Preparation

A total of 40 mg of fresh tissue of the test YSB sample and standards were processed for sample preparation. The freshly collected sample tissues were chopped into small pieces using a double-edged sharp razor blade (12 mm size) as described by Galbraith et al. (1983) in plastic Petri dishes (20 mm diameter) kept on ice, containing 1 mL of ice-cold nuclei isolation buffer [18]. A 40 μm pore size nylon cell strainer (Corning, New York, NY, USA) was used to filter the resulting homogenate into the sample tubes. The homogenate solution of approximately 2 mL was further divided into 3 aliquots of 0.5 mL each, to which 50 μg/mL PI was added for staining of the nuclei. Following this, 50 μg/mL of RNase A from a stock solution of 6 mg/mL (Sigma-Aldrich, St. Louis, MO, USA) was added to remove the RNA contamination. Samples were incubated in dark for 1 h. All the samples were kept on ice until further analysis in the flow cytometer, Bacton Dickinson FACS LSR-II (BD Biosciences, San Jose, CA, USA).

### 2.9. Flow Cytometric Analyses

Stained samples were acquired on a flow cytometer using a blue laser line (488 nm) to excite PI and a 585/42 Bypass filter to collect emission signals. Singlet G0/G1 population was analyzed for mean fluorescent intensity (MFI) of PI channel using BD FACSDiva software v8.0.1. The DNA content (2C value) in pg was calculated by using the formula: Sample DNA content (pg) = Reference DNA content (pg) X [MFI of G0/G1 (Samplepeak)/MFI of G0/G1 (standard peak)]. Results were expressed using the conversion factor 1 pg DNA = 978 Mb for genome size estimation.

### 2.10. RNA-seq Mapping

The RNA-seq reads obtained from the de novo transcriptome analysis performed at the first, third, fifth, and seventh larval developmental stages of YSB were downloaded from the NCBI SRA database (SRX733621) and mapped on the assembled genome of *S. incertulas* using BWA-Mem version 0.7.12 with parameters of minimum seed length 19, the penalty for mismatch 4, and gap open penalties for deletions and insertions—6. 

### 2.11. Evolutionary Analysis

To determine how YSB is related to other insect pests, we ran multiple sequence alignment on the single-copy gene cluster generated using OrthoVenn on the proteomes of the organisms, namely, *N. lugens, C. medinalis*, and *C. suppressalis*. The resulting alignment file was analyzed using Mr Bayes (http://www.phylogeny.fr/one_task.cgi?task_type=mrbayes, accessed on 31 January 2020). The phylogenetic tree was reconstructed using the Bayesian inference method implemented in the Mr Bayes program (v3.2.6). The number of substitution types was fixed to 6. The Poisson model was used for amino acid substitution, while rates variation across sites was fixed to ‘invgamma’. Four Markov chain Monte Carlo (MCMC) chains were run for 10,000 generations, sampling every 10 generations, with the first 250 sampled trees discarded as ‘burn-in’. Finally, a 50% majority-rule consensus tree was constructed.

## 3. Results

### 3.1. YSB Genome Size Estimation and Assembly Statistics

**Figure 1:**
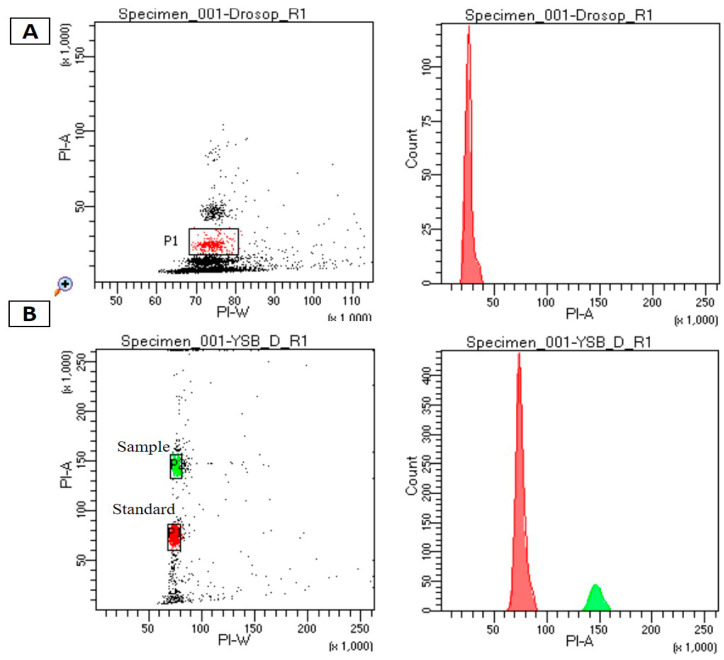
Genome size estimation through flow cytometry analysis: (**A**) histograms of propidium iodide fluorescence intensity for *Drosophila melanogaster* standard references used in the study. (**B**) Histograms of propidium iodide fluorescence intensity in *Scirpophaga incertulas* with *Drosophila melanogaster* as a standard reference.

To obtain preliminary information on the YSB genome size, we deployed flow cytometry for its genome size estimation. It is a reliable method to estimate the genome size of organisms, especially when a species or an organism is being sequenced for the first time. The genome size of YSB ranged from 347.6 to 370 Mb (Figure 1 and Table 1).

*Drosophila melanogaster* was used as a reference species. Percentage of variation within replication was 0.95–1.04% and with internal control. 1 C value is genome size of *D. melanogaster*—176.4 Mb; 1pg = 978Mb 1C value of YSB = 0.378 pg = 369.684.

The Illumina HiSeq short reads paired-end and mate-paired raw reads were subjected to quality filtering, resulting in 24.67 and 21.12 million high-quality reads, respectively. The total data for paired-end and mate-paired reads accounted for 7.3 Gb and 6.3 Gb, respectively, totalling up to 13 Gb. The de novo assembly resulted in 310,612 scaffolds with an average scaffold size of 993 bp (Table 2). The maximum and minimum scaffold sizes were recorded at 46,270 and 300 bp, respectively, with an N50 value of 1260 bp (Table S1). 

The total genome size achieved upon scaffolding accounted for 308 Mb. The estimated genome size using flow cytometry (median 356 Mb) correlated with the estimated genome size (87% in terms of genome coverage) through sequencing. The average GC content of the YSB genome was observed to be 36.37%. The completeness of draft assembly validated using CEGMA (Core Eukaryotic Genes Mapping Approach) and BUSCO (Benchmarking Universal Single-Copy Orthologs) respectively revealed 57.26% and 48.87% gene annotations matching with the total number of core genes in the database.

The data were submitted to NCBI (biosamplehelp@ncbi.nlm.nih.gov, accessed on 30 January 2020) BioSample database under the accession SAMN12500696.

### 3.2. Structural Annotation of YSB

#### 3.2.1. Protein-Coding Genes Prediction

A total of 46,057 genes were predicted from the scaffolds, with an average gene length of 427 bp. Functional annotation of these genes using blastx showed 30,306 (65.80%) genes with blast hits and the remaining 15,751 (34.19%) genes remained unannotated. The 15,751 unannotated genes did not indicate any similarity with the genes present in the existing repertoire of databases. Unannotated genes have been reported earlier in insect genomes and can be attributed to the paucity of data available for the reference genome, its completion, and assembly [12]. Among the 46,057 annotated genes, 5840 genes were assigned with Gene Ontology (GO) terms (Figure S1). From the GO analysis, molecular function made up the majority (2404) followed by biological process (2087) and cellular components (1349). Under biological processes, genes involved in the metabolic processes (GO: 0008152) and cellular processes (GO: 0009987) were highly represented. In molecular functions, catalytic activity (GO: 0003824) was the highest, followed by binding activity (GO: 0005488), whereas in cellular components, the most represented categories were cells (GO: 0005623) and cell parts (GO: 0044464). The YSB genes were mapped to reference canonical pathways in KEGG (Table S2) and were categorized into metabolism, cellular processes, genetic information processing, environmental information processing, and organismal system. Out of the total 46,057 predicted genes, a total of 5230 genes were annotated with the KEGG pathway. The most enriched KEGG pathways were signal transduction processing, transport and catabolism process, endocrine system, carbohydrate metabolism, and cell growth and death process. A total number of 19,113 sequences were assigned to the COG classifications (Figure S2). Among the 24 COG categories, the cluster for function unknown (5226, 27.34%) was the highest, followed by signal transduction (3028, 15.84%), transcription (1307, 6.83%), post-translational modification, protein turnover, and chaperone functions (1295, 6.77%).

#### 3.2.2. Non-Coding RNAs (ncRNAs)

The non-coding RNAs (ncRNAs) are important regulatory molecules, especially at the post-transcriptional level [19]. Several types of ncRNAs, including ribosomal RNA (rRNA), transfer RNA (tRNA), small nuclear RNA (snRNA), small nucleolar RNA (snoRNA), microRNA (miRNA), and long non-coding RNA (lncRNA), were annotated through Rfam database, and the details of each class are mentioned in Table S3. In addition to this, using a homology-based search through blastN against rnammer 1.2 database, 9273 tRNAs were predicted (3211 cove-confirmed tRNAs and 2174 tRNAs with introns).

#### 3.2.3. Transposable Elements

Transposable elements (TEs) are fundamental to the evolution and expansion of insect genomes. The YSB genome was found to have about 1% of TEs. Among the TEs, DNA elements represented the highest number (1500), followed by SINEs, LINEs, LTR elements, non-LTR retrotransposons, retrotransposons, and other transposable elements. The transposable elements namely hAT-Charlie, TcMar-Tigger, piggyback were predominantly present in the DNA element whereas and Mobile element jockey-like, were more in non-LTR retrotransposon categories (Table S4). 

### 3.3. Functional Annotation

#### 3.3.1. Genes Governing Body Parts

We identified several organ developmental genes and categorized them according to the YSB body structure (Table S5). These include antennapedia complex, bithorax complex, *Hox* cofactors, *Hox* protein-regulating genes, cuticle proteins, different segments of the antenna, retinal determination network, wing development, and homeobox genes. These genes, along with the other predicted genes, might control the organ development of the YSB, as evidenced by several other studies. 

#### 3.3.2. Gene Regulation in YSB

We deduced three levels of gene regulation in YSB, namely, transcription factors, hormones, and epigenetic mechanisms.

##### Transcription Factors (TFs)

Several TFs such as zinc fingers, helix–turn–helix (HTH) classes containing homeodomain transcription factors, forkhead box TFs, and heat shock factor (HSF) TFs, basic DNA-binding domain TFs that include basic helix–loop–helix (bHLH), basic leucine zipper (bZIP), hormone receptors, and Myb isoforms were identified from the YSB genome, the largest being the zinc finger (391 in number) with different domains. Hox genes (homeobox-containing transcription factors) are typically found in clusters within the genome, and it has been suggested that the evolution and expansion of Hox genes have played a key role in region-specific identity during early development in the majority of insects. Other homeobox TFs (*araucan* and *caupolican*-like belonging to *Iroquois* complex; *Iro-C*) were also found in YSB and have been reported for controlling proneural and vein-formation in Drosophila [20].

The *SOX* transcription factors (belonging to the high-mobility group (HMG) superfamily of DNA-binding proteins) were found to be in high number (11). In addition to this, larval metamorphosis, vitellogenesis, and oocyte maturation controlling methoprene-tolerant (Met) gene that has a basic helix–loop–helix (bHLH) domain was detected in YSB as reported from other insects such as cotton bollworm (*Helicoverpa armigera*) [21,22,23]. The YSB also has Krüppel homolog 1 (Kr-h1), a zinc-finger transcriptional factor that is known to play a pivotal role in ovariole development and egg maturation in *N. lugens* [24].

Several BTB (bric-a-brac, tramtrack, broad complex transcription regulators) TFs (BTB-only proteins, BTB-ZF proteins, BTB-Kelch proteins, and BTB-ANK protein) originally identified in *D. melanogaster* that are known to be involved in the cross-talk between the signaling pathways of juvenile hormone and ecdysone were also observed in YSB [25,26]. The circadian clock seems to be regulated by clock work orange (CWO) TF, belonging to the basic helix–loop–helix ORANGE family, as reported in Drosophila, was also detected in the YSB genome [27]. Thus, the identified TFs in YSB may be involved in the regulation of various physiological procedures right from organ development to signal transduction. The detailed list of YSB TFs is found in Table S6.

##### Hormonal Regulation

Insect molting and metamorphosis are regulated by steroid molting hormones and juvenile hormones (JHs), respectively. The key genes associated with the JH biosynthesis pathway and hormones associated with molting were principally observed under this category (Table S7). The phylogenetic analysis of the ecdysone receptor of YSB clustered separately from the other species (Figure S3) indicates its diversity. The phylogenetic analysis of the pheromone-binding gene of YSB clustered with *H. armigera* separately from the other insect species (Figure S4). Additionally, vitellogenin (Vg) and vitellogenin receptor (VgR), which are known to provide nutrients for the embryonic development in several insects [28], were also found in YSB. Adipokinetic hormone receptor reported in *N. lugens* for promoting vitellogenin uptake by oocytes [29] also seems to be operative in YSB. The phylogenetic analysis of Vg gene of YSB was clustered with navel orange worm (*Amyelois transitella),* a pest that belongs to the lepidopteran order (Figure S5).

##### Epigenetic Mechanism

Genomic profiling reveals the presence of several genes required for DNA methylation and histone modification (Table S8). Among these, histone-lysine N-methyltransferase, histone deacetylase, a histone acetyltransferase, histone lysine demethylase, site-specific DNA-methyltransferase, and DNA cytosine-5 methyltransferase were identified from the data.

### 3.4. YSB as a Monophagous Rice Pest

The genome data sheds light on the monophagous nature of YSB towards its host plant. It has well-equipped three-tier mechanisms related to vision, chemosensory, and digestion that assist in searching, recognizing, and successful colonization of its host.

#### 3.4.1. Visual Perception

The YSB is a dynamic lepidopteran that migrates over a long distance in search of rice plants. Visual stimuli in insects are principally mediated by opsin protein, which is a member of the G protein-coupled receptor family [30]. Opsin genes allow the insect to visualize according to the environmental cues, photoperiod, light sensitivity, and humidity. The number of genes for visual perception varies according to the behavioral characteristics of insect species. A total of 17 copies belonging to several types of opsin genes were found (Table S9). The phylogenetic analysis of the long-wavelength opsin gene of YSB clustered separately away from the other species (Figure S6). Along with opsins, 43 members of G protein-coupled receptors (GPCRs) were also identified from the YSB genome. GPCRs are the largest family of cell membrane proteins that are activated by a wide variety of ligands (light, ions, neurotransmitters, odors, and hormones) [31]. The unique gene repertoire might allow YSB to perceive the signals and identify the rice host specifically by avoiding the non-host species, thus becoming a strictly monophagous pest.

#### 3.4.2. Chemosensation

In addition to visual perception, the YSB engages strong chemosensory genes (140 genes) for olfaction that might be operated through major odorant-binding proteins (OBPs). Insect OBP genes have been identified throughout Neoptera class [32,33]. One copy of odorant-degrading enzyme (ODE), 21 copies of odorant receptors (ORs), along with 43 members of ionotropic glutamate receptors (IRs) and 3 sensory neuron membrane proteins (SNMPs) seem to participate in the olfaction machinery of YSB. Apart from these, other families of chemosensory gene families included 4 gustatory receptors (GRs), 1 gustatory and odorant receptor, 15 chemosensory proteins, 1 chemosensory ionotropic receptor, and 13 metabotropic glutamate receptors (Table S10), which were also identified. Interestingly, metabotropic chemosensory receptors reported to be exclusively used by vertebrates [34] were also found in YSB, and this gene had less homology compared to other insect species (Figure S7). 

#### 3.4.3. Digestion

Complex digestive enzymes also seem to be operative in YSB (Table S11), as in other lepidopteran insects. Aminopeptidase N (APN) is a midgut enzyme in a majority of insects including YSB. APN of YSB showed high sequence similarity with the diamondback moth (*Plutella xylostella*) (Figure S8). Serine proteases (SPs) are another class of digestive enzymes known to be involved in digestion, development, and innate immunity in several insects [35,36]. However, phylogenetic analysis of serine protease of YSB clustered distinctly from the other insects, showing that it is distinctively evolved, whereas alpha-amylase of YSB clustered with *T. castaneum*, which might be conserved among Insecta class (Figures S9 and S10). 

### 3.5. Defensive Mechanisms in YSB

#### 3.5.1. Metabolic Detoxification

Insects have evolved specialized detoxification mechanisms to cope with plant defenses [37]. A wide range of detoxifying genes (ABC transporters, members of ATP-binding cassette sub-family, cytochrome P450, cytochrome P450 monooxygenase, glutathione S-transferase, carboxylesterase, cholinesterase, and UDP-glycosyl transferase) associated with metabolic detoxification was detected in YSB, which indicates that it has a well-evolved detoxification mechanism (Table S12). Cytochrome P450 (CYPs) genes predicted to be involved in metabolic detoxification and inactivation of endogenous toxic compounds were reported in several insects [38,39]. In the present study, we found 130 genes that belong to different families of CYP 450 in the YSB genome. Similarly, six subclasses of GST (one delta, four epsilon, two omega, three theta, one zeta, and two sigma) were also identified in YSB, potentially being involved in the detoxification of both endogenous and xenobiotic compounds such as those observed in other insects. Nine GST genes were reported in *S. furcifera* and 13 were found in *N. lugens* [40]. Comparatively, the presence of a higher number of detoxifying genes in YSB over other insect pests implies that YSB might develop quick insecticide resistance (Table 3). Although reports on insecticide resistance in this pest are sparse, a confirmed report of diamide resistance in *Scirpophaga* (species not specified) from Indonesia through lab assay and poor field efficacy was reported [41].

#### 3.5.2. Circadian Genes

The behavioral activities in the majority of insects such as locomotion, courtship, mating behavior, seasonal adaptations, egg-laying, and photoperiodism mainly depend on the circadian rhythms. Circadian rhythms are particularly important for timing or regulating key biological events in insects [44]. In YSB genome data, circadian-regulated genes, namely, timeless, circadian clock-controlled-like, clock, cycle protein, and cryptochromes including CRY 1 and CRY 2 along with other *takeout* genes and *takeout JHBP*-like homologs were identified (Table S13). 

### 3.6. Behavioural Activities of YSB

#### 3.6.1. Cacophony

Cacophony (cac) is a phenomenon of exchanging the signal patterns between two different sex types during courtship. It was reported to be controlled by *cac* gene, which encodes an α-1 subunit of a voltage-dependent calcium channel [45,46,47]. Interestingly, *cac* gene was also found in YSB (Table S14), indicating the presence of this kind of behavior. Phylogenetic analysis of the *cac* gene shows the highest similarity to *Danaus plexppus*, which signifies that *cac* activity is a conserved phenomenon among a few Lepidopteran insects (Figure S11). Along with this, several other reproduction-related genes are also identified from YSB (Table S15).

#### 3.6.2. Gravitaxis

The movement of insects towards gravity or opposite to gravity was observed, which is known as gravitaxis. This kind of phenomenon initially observed in Drosophila and subsequently in some other insects, is known to be regulated by the *alan shepard* (shep) gene. Two gene members coding for alan shepard (*shep*) were found in the YSB genome (Table S14) and were found to be more homologous to the *shep* gene of *N. lugens* (Figure S12). 

#### 3.6.3. Anaerobic Environment 

Regulation of tissue responsiveness to oxygen deprivation is known to be controlled by the *anoxia upregulated* gene. Interestingly, two *anoxia upregulated* genes showing sequence similarity with *H. armigera* (Figure S13) were identified from the YSB genome, which might play role in adapting to the flooded field condition during the diapause stage. We presume that both the genes coding for gravitaxis and anoxia seemed to work concurrently in YSB, which could reflect the novel behavior of YSB during infestation in rice. 

#### 3.6.4. Serotonin Receptor

Serotonin or 5-hydroxytryptamine (5-HT) is a biogenic monoamine found across most phyla of life [48]. Serotonin activates its receptors that belong to the families of G protein-coupled receptor (GPCR) and the ligand-gated ion-channel. A total of 12 serotonin receptors and 4 sodium-dependent serotonin transporters were found in YSB, which may function similarly. It also provides a clue in terms of insect recognition of the host, rice. The phylogenetic analysis of the serotonin receptor gene (YSB_Gene5911_Sccafold_6479) of YSB clustered quite apart, leaving all the other insect species, making it unique in its behavioral activities (Figure S14).

### 3.7. Genomics-Assisted Management of YSB

#### 3.7.1. Insecticide Targets

YSB genome has enabled us to detect the reported targets (nicotinic acetylcholine receptor (nACHR), γ-aminobutyric acid receptor (GABA), glutamate-gated chloride channel (GluCl) and voltage-gated sodium channel (VGSC), acetylcholinesterase (AchE), acetyl-CoA carboxylase (ACCase), cys-loop ligand-gated ion channel (cysLGIC) superfamily and ryanodine receptor (RyR)) for insecticides (Table S16); expression of some of them were reported in the transcriptome study [13]. The phylogenetic analysis of one of the target genes, GABA, from YSB appears to be distinct compared with other insect species; this specificity might be explored for employing controlling strategies of YSB (Figure S15). 

#### 3.7.2. Immunity Genes

The innate immune system is the first line of defense against invading pathogens, which generally operates on a minimal scale in insects, unlike vertebrates [49]. However, pattern recognition receptors (PRRs) were reported in insects, which helps in the recognition of pathogens. One of the PPRs, peptidoglycan recognition protein (PGRP), was well characterized in silkworm, which binds to peptidoglycan of Gram-positive bacteria and triggers polyphenoloxidase (PPO), activating cascade [50]. YSB has five PPOs genes in the genome. βGRPs are also reported to have a strong affinity to β-1, 3-glucan of fungi, and lipopolysaccharide (LPS) of Gram-negative bacteria. Interestingly, several reported PRRs such as Immulectin 1-4 (IML1-4), seven copies of peptidoglycan recognition protein 1 (PGRP1), nine copies of β-1,3-glucanase-related protein 1-3 (βGRP1-3), c-type lectins (CTLs), galectins, leucine-rich repeat proteins (LRRPs), hemocytins, toll-like receptor, scavenger receptors (SCRs), and Down syndrome cell adhesion molecule (DSCAM) were detected from the YSB genome, which might have a similar role in imparting immunity (Table S17). Six copies of apyrases (Apyrases are salivary proteins reported predominantly in blood-feeding arthropods *A. aegypti* and *Anopheles albimanus*), known to inhibit the platelet aggregation [51,52], were found in YSB. In addition to the above-described immune-related genes, 6 attacin precursors and 4 attacin-like proteins (glycine-rich antimicrobial polypeptides), 1 copy of gloverin, 3 copies of galectins, and 15 copies of lysozyme were also found in YSB. Insects also have a different defense mechanism towards viruses by employing the toll and IMD pathways [53,54,55]. Several components involved in the toll pathway (1 cactus, 1 spaetzle, 2 pellino, 2 cactin-like isoforms, and 2 dorsal) and a few IMD pathway genes were identified from the YSB genome, indicating that YSB also uses the toll pathway for resistance towards viruses. It was observed that YSB has a well-built innate immune system, including the presence of immunity regulators such as serpins (serine proteinase inhibitors) and serine proteinase homologs (SPHs) (Table S17), which perhaps makes the biological control of YSB tougher compared with other rice pests.

#### 3.7.3. Insect Effectors

When insects attune on the plants for their endurance, their contact on the plant surface elevates the plant defense mechanisms with the concomitant production of secondary metabolites such as alkaloids, glucosinolates, terpenoids, and phenolics [56]. In response to plant defenses, insect pests use their effector/s arsenal to suppress plant immunity [57,58]. Upon mining the effector-like proteins in the YSB genome, we found 36 different venom-related genes that might work as potential effectors (Table S18), which seem to suppress the rice immunity, thus making susceptible reactions in many rice genetic resources. The venom gene of YSB appeared to be distinct from the other insect species, specifying its uniqueness of employing precise effectors in defeating the host immunity (Figure S16). Further, the characterization of the effector arsenal of YSB will provide novel and significant information on its interaction with rice, which may lead to insights for pest management strategies.

#### 3.7.4. RNAi Machinery

In insects, three major types of RNA silencing pathways were majorly studied, namely, microRNAs (miRNAs) involved in the regulation of gene expression [59], small interfering RNA (siRNAs) involved in anti-viral defense, and PIWI-interacting RNAs (piRNAs) involved in defense against transposons transposition in the germline [60]. The existence of RNAi machinery in YSB was known from transcriptome study [13], but in the genome data, we identified complete RNAi machinery, which includes RNaseIII, Dicer-1, Argonaute-1, Argonaute-2, Piwi, SID, exportin, epsin, liquid facet, and *sid*-1 (Table S19). 

### 3.8. Genomic Resources

We evaluated the entire genome for SSR (single sequence repeats) prediction and found 19,637 (6.3%) sequences comprised of 21,696 SSRs motifs. More than one SSR was found in 1784 scaffolds (Table 4). 

**Figure 2:**
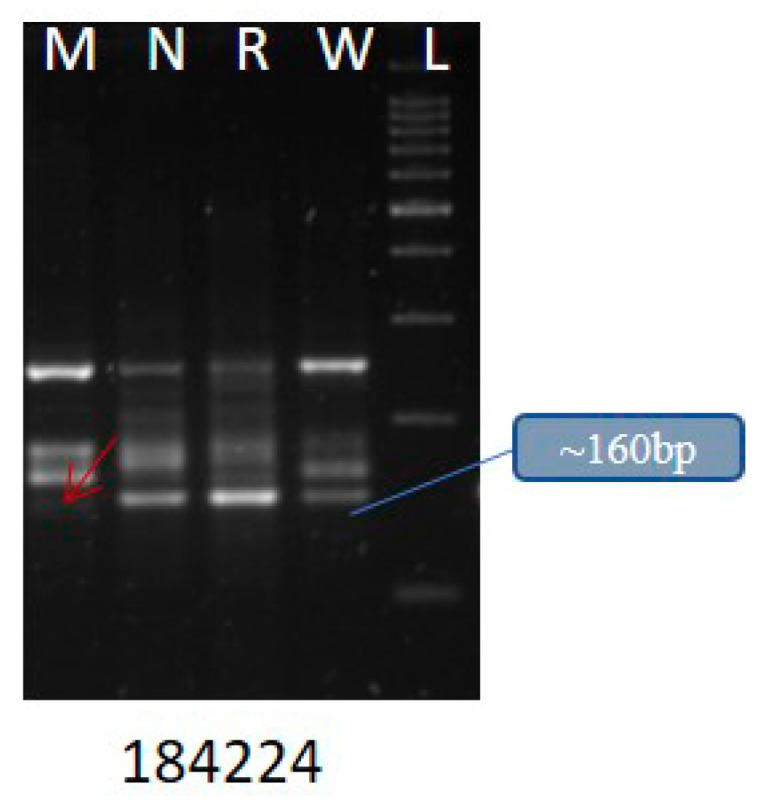
Representative SSR showing polymorphism among the natural. populations of YSB from four different locations: Medak (M), Nizamabad (N), Rajendranagar (R), and Warangal (W) of Telangana state.

We found that di- and trinucleotide SSRs (62.2% and 24.1%, respectively) were significantly higher than other repeat types reported from the other insects [61]. Among the di- and trinucleotide classes, AT/AT (75.41%) and AAT/ATT (45.87%) were predominant. We also validated 25 SSRs (Table S20) with 4 different YSB populations collected from Medak, Nizamabad, Rajendranagar, and Warangal and found differences among the populations (Figure 2). We observed that the gSSR, namely, YSB_SSR_184224 coding for gustatory receptor showed polymorphism. The allele’s number ranged from one to four. 

### 3.9. Mapping the Transcriptome Data to the Genome

In our earlier study, we documented the transcriptome changes that occurred at four larval stages through RNA-seq. Around 88.32% of RNA-seq reads matched to the de novo assembled scaffolds (Table 5), indicating that the majority of expressed genes were assembled to the genome. The annotated transcripts were also compared with the gene annotated from the genome data, which revealed that most of the annotations were similar. We compared the transcripts expressed in the RNA-seq data with the genes based on its function. Most of the transcripts were represented in the genome, except a few that may be very much specific to the larval stages. The transcripts such as Yokozuna protein, which is a Ty1/Copia LTR retrotransposon categorized for the first time in *B. mori* genome [62], were expressed at the L1 stage of YSB transcriptome. The cuticular precursor, aminopeptidase N (*APN*), which has a role in dietary protein digestion, was expressed at the L5 stage of YSB transcriptome [13]. Carboxylesterase, which is involved in a variety of physiological functions such as degradation of neurotransmitters and metabolism of specific hormones and pheromones, and subsequently involved in insect development and behavior [63] at L7 stage from the transcriptomic data generated by using various YSB larval stages [13], was also found in YSB genome data.

### 3.10. Comparative Genomic Analysis

**Figure 3:**
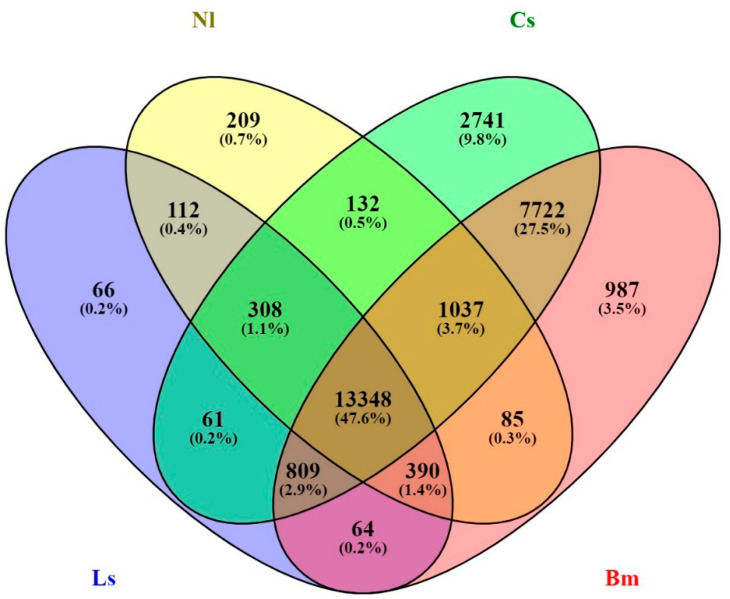
Venn diagram depicting the relation between four different insect species. The Venn diagram shows the common and unique genes of *N. lugens, C. suppressalis, L striatellus*, and *B. mori* shared with YSB.

The genome-level relatedness of YSB with four insects, namely, *C. suppressalis, N. lugens, B. mori,* and *L. striatellus* belonging to two Lepidopteran and Hemipteran orders were analyzed. The Venn diagram showed the number of shared and unique genes in YSB. The YSB shared 26,158 genes (56.79%) with *C. suppressalis*, 24,442 genes (53.06%) with *B. mori*, 15,621 genes (33.91%) with *N. lugens*, and 15,158 genes (32.91%) with *L. striatellus*. A sum of 13,348 genes was conserved among all four insects, which might be important for the Neoptera group. YSB shares 2741 genes solely with *C. suppressalis*, which demonstrates its closeness; 209 with *N. lugens*; 987 with *B. mori*; and only 66 genes were shared between YSB and *L. striatellus*. A total of 308 genes were commonly shared among *C. suppressalis, N. lugens,* and *L. striatellus* (Figure 3). A total number of 2979 SNP variations (1838 homozygous SNPs and 1141 heterozygous SNPs) were found between the genomes of YSB and *B. mori*.

### 3.11. Evolutionary Timeline Analysis of YSB and Three Major Rice Pests 

The phylogenetic analysis of YSB was performed with the other three rice pests (*N. lugens, C. medinalis*, and *C. suppressalis*) and showed that YSB evolved closely with *N. lugens* followed by *C. medinalis and C. suppressalis* (Figure S17). 

## 4. Discussion

The draft genome assembly of YSB is larger than a few Lepidopteran genomes such as monarch butterfly (*Danaus plexippus*)—248 Mb [64], postman butterfly (*Heliconius melpomene*)—266 Mb [65], swallowtail butterfly (*Papilio polytes*)—227 Mb [66], and also with the reference genome used in this study *Tribolium castaneum* (204 Mb), and smaller than other rice pests such as Asiatic rice borer or striped rice stem borer (*C. suppressalis*)—824 Mb [67], fall armyworm (*Spodoptera frugiperda*)—371 Mb [68], brown planthopper (*N. lugens*)—1140 Mb [69], and small brownhopper (*L. striatellus* Fallen)—541 Mb [43]. The differences in the genome size may arise due to the presence of repetitive elements in the genome and other factors such as physiology and environment [70]. The GC content of the YSB genome was observed to be similar to other rice pests such as *N. lugens* (37.59%) [70], *L. striatellus* (34.5%) [43], *S. furcifera* (31.6%) [12], and *S. frugiperda* (32.97%) [71], whereas common fruit fly (*Drosophila melanogaster*) has a GC content of 43% [72]. Since this is the first maiden effort to sequence the YSB genome, the genome assembly coverage (CEGMA and BUSCO) was comparatively lower. Flow cytometry provides a priori information on the genome size and is a prerequisite for new sequencing projects. Subtle differences in the genome size of some pests such as small brown planthopper (*L. striatellus*), white-backed planthopper (*S. furcifera*), and N. *lugens* upon flow cytometry were observed to be 587, 733, and 1110 Mb, respectively, whereas the final genome assemblies were 541, 720, and 1140 Mb, respectively [43,70,73], which could be insignificant.

The non-coding RNAs play a crucial role in gene regulation, and presence in YSB also suggests the same. A further level of investigation is required for deciphering regulatory functions of specific ncRNAs along with their potential relationships. Interactions among different ncRNAs will provide a clear picture of gene regulation through ncRNAs. Transposable elements in insect genomes range from as little as 1% in the Antarctic midge [74] to as high as 65% in the migratory locust [75]. The YSB genome has about 1% of TEs, which is significantly less compared with other insect pests such as red flour beetle (*Tribolium castaneum;* 6%) [76], *B. mori* (35%) [77], *S. frugiperda* (20.28%; the highest are SINEs at 12.94%)*, N. lugens* (38.9%; majority are LINES (16%)) [70], *S. furcifera* (39.7%, with 17.33% of DNA transposon as largest class) [73]. A lower percentage of TE in the YSB genome may be due to TE extinction, which needs to be further studied in detail, or it could be due to a draft assembly and is likely to improve as the assembly is upgraded further. 

The transcription factors (TFs) are involved in several biological processes that work to ensure the appropriate expression of genes at a specific time at specific tissues in organisms. The zinc finger TFs were also observed in earlier-reported RNA-seq data at larval stages of YSB [13] and several other insects such as *B. mori* [78], implying the importance of ZFs. The role of these SOX TFs as transcriptional activators or repressors in *D. melanogaster* were reported [79,80,81]. However, the number is high in YSB as compared to other insects such as the western honey bee (*Apis mellifera*), red flour beetle *(T. castaneum*), jewel wasp (*Nasonia vitripennis*), and fruit fly (*D. melanogaster*) [82,83,84].

Hormonal regulation is critical for insects, and specific hormones can be targeted to control the pest infestation. The silencing of Vg and VgR has been successfully demonstrated for controlling *S. furcifera* and *N. lugens* [85,86]. Thus, the Vg and VgR genes identified from YSB could serve as potential targets for pest control strategies. The phylogenetic analysis of Vg genes implied the sequence is conserved among the lepidopterans and might function similarly. Studying these genes in depth may reveal the hormonal regulations in YSB, which in turn will also be used in analyzing other lepidopteran pests. 

The occurrence of many epigenetic-associated genes in YSB confirms the existence of a typical insect epigenetic regulatory structure. The regulations of epigenetic mechanisms have not yet been exploited, except in a few studies, for instance, fecundity in *N. lugens* was sharply lowered after knockdown of Dnmt1 [87]. Similarly, sexual phenotypic variations in *S. furcifera* were also related to DNA methylation [88]. The presence of a higher number of opsins (17) as compared to other insects, namely, mosquitoes, contain 8–9 [89]; *T. castaneum* contains only two; and *D. melanogaster* contains seven [76,90], and the presence of unique long-wavelength opsin gene in YSB determines its specificity in the visual mechanism. The YSB has specialized and unique visual, chemosensory, and digestive machinery, which makes it distinct from the other insect pests in choosing its host precisely. The presence of distinct olfactory genes in YSB indicates its monophagous nature and is in concurrence with the previous reports of *N. lugens* genome [70] (Table 6). Among the digestive enzymes, serine proteases of YSB were different from the other insects, indicating its distinctness for feeding behavior. Presence of circadian rhythm genes such as cryptochrome1 (CRY1), a blue-light photoreceptor, and cryptochrome2 (CRY2) were detected in YSB, as was reported in other insects such as mosquitoes and butterflies [91,92]. The CRY1 gene was found in *D. melanogaster* [93], whereas CRY2 was found in *A. mellifera* and *T. castaneum* [91,94], which correlated with the finding that all Lepidopteran species harbor two *cry* genes, indicating to some extent the correctness of our draft genome assembly. The *Takeout* family genes are another class discovered as a circadian-regulated gene found in the black-brown fly (*Phormia regina*) [95] and tobacco hornworm (*Manduca sexta*) [96]. These genes together might play a role in circadian pathways operating in YSB. 

The behavioral genes such as cacophony (*cac*), *alan shepard* (shep), and *anoxia upregulated*, which were detected initially in *D. melanogaster* [45,46,47,104,105], also seemed to be functional in YSB. These genes also play a role in courtship, adult locomotory behavior, and metamorphosis in other insect species [106]. Serotonin receptors, which play a crucial role in physiological and behavioral processes in insects and other invertebrates [107], were found in the YSB genome. Several experiments on different insects have demonstrated their role in vision [108], olfaction [109], hearing [110], feeding [111], circadian behavior [112], and learning and memory [113,114].

Several insecticide targets were identified including members of the cysLGIC superfamily and nicotinic acetylcholine receptors. The cysLGIC superfamily genes are known to mediate chemical synaptic transmission in many insects and have been effectively targeted by various chemicals for crop protection strategies to date [115]. The nicotinic acetylcholine receptors (nACHR) in YSB harbored eight α and two β subunits similar to other insect genomes such as *A. mellifera* (9 α and 2 β subunits), *T. castaneum* (11 α and 1 β subunit), and *N. vitripennis* (12 α and 4 β subunits) [116]. On the basis of this information of insecticide targets, new molecules can be designed for targeted YSB control.

Insect immune defense genes are an arsenal for host pest infestation and are critical for pest control. Several immune-related genes were present in YSB, among these, seven PGRPs were detected, whereas two PGRPs and seven βGRPs were reported in *N. lugens* [69]. The polyphenol oxidase PPO is also one of the major innate immunity proteins involved in cellular and humoral defense [117,118]. Foregut PPO in *D. melanogaster*, *B. mori*, and *H. armigera* was reported as the main factor for detoxification of plant phenolics. If these genes are lost, the metabolism of plant phenolics will be impaired, and these may become toxic to the insects [119]. Functional roles of attacins were discovered in the silk moth (*Hyalophora cecropia*) [120], *B. mori* [121], and a few other lepidopteran and dipteran species [122]. However, attacins and gloverins were reported to be absent in the *N. lugens* genome. Apyrases are known to limit the plant eATP accumulation by hydrolysis of ATP, which was made evident in the case of *Helicoverpa zea* on tomato [123]. Consequently, the YSB might be employing apyrases to reduce the plant eATP availability, which might be leading to aberrant plant growth and development.

We found venom-related genes might also work as the potential effectors in YSB, along with other effectors such as oral secretion, digestive enzymes, alkaline phosphatase [124], and β-glucosidase [125] which were previously in other insects. Recently, the HARP1 gene was reported to act as an effector from the oral secretion of the cotton bollworm (*H. armigera*). Similarly, R-like/venom proteins from parasitoid wasps (*N. vitripennis* and *Trichomalopsis sarcophagae*) and stink bug (*Pristhesancus plagipennis*) were reported to function as effectors [126]. The presence of RNAi machinery confirms the occurrence of systemic spread of dsRNAs through *sid*-1. Thus, the data provide strong evidence about the possibility of employing RNAi as an effective controlling strategy. Recently, our team has demonstrated the knockdown of the acetylcholinesterase (AChE) gene of YSB through RNAi as a pest control strategy [127].

The evolutionary closeness of YSB with *N. lugens* might be due to the similarity in feeding behavior (monophagous rice sapsucker). The divergence with other rice pests may be admitted to the behavioral genes in addition to the effectors, immune system, etc.

We used four representative natural populations for diversity studies using genomic SSRs. The polymorphism between YSB populations was comparatively less and might be due to the differences in the levels of insecticide exposure, external pressure, etc. Various biological traits and genetic diversity might associate with populations from different geographic localities due to geographic isolation, influenced by different climatic effects and thus different selection pressures [128]. Microsatellite markers have also been used in studies of hymenopteran sex ratios to identify the sex of eggs [129,130] for the detection of genetic variation between Goniozus wasp populations [131]. The YSB genomic SSRs can be utilized for diversity studies of a higher number of YSB populations collected from different ecological and geographical regions.

## 5. Conclusions

The unavailability of the YSB genome sequence thus far has been hampering efforts to gain a complete hold on the YSB management strategies. The present draft genome sequence reveals that, though it has a small genome size, it uses its entire genomic information perfectly. The genome information provided a snapshot of complex metabolic mechanisms that operate in the insect to manifest and evolve in nature. We not only observed the different gene sets but also observed that YSB has a high level of gene regulation by operating a repertoire of transcription factors, hormonal control, and epigenetic mechanisms. YSB uses a strong visual and chemosensory mechanism to become monophagous. The genome analysis led to the identification of genes related to its specific behavior. Comparative genomic analysis with other rice pests (*C. suppressalis, N. lugens*, and *L. striatellus*) and non-rice pests (*B. mori*) reveals that YSB is unique in the way it has evolved based on its visual perception, digestive enzymes, and presence of RNAi machinery. The presence of a high level of immune receptors and complete RNAi machinery gives us reasons for the non-functioning of viral suspensions on YSB. The presence of diverse effectors and their possible use by the insect provide a ray of hope to the biologist to exploit the mechanisms of these molecules for insect control. The information emanated from genome analysis matched the earlier reported transcriptome profiles and found that this information is at a much higher level than the transcriptome data. The identified SSR markers are a better genomic tool in understanding the diversity of populations/biotypes across the globe. We have added genomic SSRs to the existing repertoire in addition to our earlier EST SSRs, making them a rich source for molecular characterization of YSB populations. These SSRs can be deployed for studying the genetic diversity, evolutionary dynamics, repetitive sequence dynamics, and population dynamics of YSB populations, their related insect pests, and other insect pests with fewer genomic resources. Overall, we made a maiden effort to determine its genome size and sequence to a significant level of an agriculturally important pest, YSB, that affects the yield of rice adversely across the rice ecosystems. Our genome and transcriptome resources of YSB helps in deciphering various “omics” of this pest, leading to a better understanding of the systems biology. Our data can be mined by both the academicians and industry in developing novel management strategies and designing a new class of safer and specific insecticide molecules.

## Figures and Tables

**Table 1:** Mean average value detected in flow cytometry.

	Mean Fluorescence Values (with Internal Control)		Genome Size of YSB (Mb)
Species	*D. melanogaster*	*S. incertulas*	
R1	73,073	145,138	347.6 Mb
R2	78,370	156,105	348.58 Mb
R3	21,116	43,991	370 Mb
R4	21,140	44,633	369 Mb

**Table 2:** Genome statistics.

No. of Scaffolds	310,612
Total no. of bases (bp)	308,440,134
Average scaffold size (bp)	993
Scaffold N50 (bp)	1260
L50 sequence count	71,684
Max scaffold size (bp)	46,270
Min scaffold size (bp)	300
GC content (%)	36.37

**Table 3:** Comparative analysis of detoxifying genes.

Species	Cytochrome P450s (CYPs)	Glutathione-S-Transferases (GSTs)	ATP-Binding Cassette Transporters (ABC Transporters)	Carboxyl/Cholinesterase (CCEs)	UDP-Glycosyl Transferases (UGTs)	References
***Scirpophaga incertulas***	134	27	144	31	10	
***Acyrthosiphon pisum***	80	28	113	29	-	[42]
***Laodelphax striatellus***	76	29	73	54	26	[43]
***Sogatella furcifera***	66	28	74	41	19	
***Nilaparvata lugens***	79	27	87	61	19	

**Table 4:** Genomic SSR statistics.

YSB Microsatellite Search Results	
Total number of sequences examined	310,612
Total size of examined sequences (bp)	308,440,134
Total number of identified SSRs	21,696
Number of SSR-containing sequences	19,637
Number of sequences containing more than 1 SSR	1784
Number of SSRs present in compound formation	1147

**Table 5:** RNA-seq statistics.

Description	*Scirpophaga incertulas*
No. of reads	118,228,459
Total data in Gb	23.6

**Table 6:** Comparison of chemosensory genes.

Feeding Pattern	Insect Species	Odorant Receptors (OR)	Odorant Binding Proteins (OBP)	Gustatory Receptors (GR)	Ionotropic Receptors (IR)	Chemosensory Proteins (CSP)	References
Monophagous	***Scirpophaga incertulas***	21	19	18	43	16	-
Monophagous	***Nilaparvata lugens***	44	10	4	44	27	[43]
Oligophagous	***Acyrthosiphon pisum***	79	15	77	15	10	[97,98]
Oligophagous	***Bombyx mori***	64	44	14	25	19	[99,100]
Oligophagous	***Danaus plexippus***	64	32	47	27	34	[64,101]
Polyphagous	***Drosophila melanogaster***	62	66	68	66	4	[99,102]
Polyphagous	***Apis mellifera***	170	21	10	-	6	[103]
Polyphagous	***Tribolium castaneum***	264	49	219	72	19	[76]
Polyphagous	***Sogatella furcifera***	39	14	5	22	24	[43]
Polyphagous	***Laodelphax striatellus***	106	16	12	38	31	[43]
Polyphagous	***Spodoptera frugiperda***	69	51	230	43	22	[68]

## Data Availability

The *Scirpophaga incertulas* genome assembly has been deposited in the National Center for Biotechnology Information (NCBI) as BioProject PRJNA558851 (https://www.ncbi.nlm.nih.gov/bioproject/?term=PRJNA558851, accessed on accessed on 30 January 2020). All data generated and analyzed during this study are included in this published article and its Supplementary Information Files.

## Supplementary Materials

The following are available online at https://www.mdpi.com/article/10.3390/insects12060563/s1, Figure S1: Gene Ontology Analysis, Figure S2: COG class distribution for YSB sample, Figure S3: Phylogenetic relationships of the Ecdysone receptor gene in Scirpophaga incertulas compared to other insects, Figure S4: Phylogenetic relationships of the Pheromone binding gene in Scirpophaga incertulas compared to other insects, Figure S5: Phylogenetic relationships of the Vitellogenin gene in Scirpophaga incertulas compared to other insects, Figure S6: Phylogenetic relationships of the Long wavelength opsin gene in Scirpophaga incertulas compared to other insects. Figure S7: Phylogenetic relationships of the Metabotropic chemosensory receptor gene in Scirpophaga incertulas compared to other insects, Figure S8: Phylogenetic relationships of the Aminopeptidase N gene in Scirpophaga incertulas compared to other insects, Figure S9: Phylogenetic relationships of the Serine protease gene in Scirpophaga incertulas compared to other insects, Figure S10: Phylogenetic relationships of the Alpha-Amylase gene in Scirpophaga incertulas compared to other insects, Figure S11: Phylogenetic relationships of the Cacophony gene in Scirpophaga incertulas compared to other insects, Figure S12: Phylogenetic relationships of the Alan Shepard gene in Scirpophaga incertulas compared to other insects, Figure S13: Phylogenetic relationships of the Annoxia gene in Scirpophaga incertulas compared to other insects, Figure S14: Phylogenetic relationships of the Serotonin receptor gene in Scirpophaga incertulas compared to other insects, Figure S15: Phylogenetic relationships of the Gamma-aminobutyric acid (GABA) gene in Scirpophaga incertulas compared to other insects, Figure S16: Phylogenetic relationships of the Venom gene in Scirpophaga incertulas compared to other insects, Figure S17: Evolutionary timeline analysis, Table S1: Scaffold Length Distribution, Table S2: KEGG Pathway Analysis, Table S3: Non-coding RNAs, Table S4: Summary of transposable elements in the YSB genome, Table S5: Body Structural Genes, Table S6: YSB Transcription factors, Table S7: Hormonal Development, Table S8: Epigenetic Genes, Table S9: Visual Perception, Table S10: Chemosensory Receptors, Table S11: Salivary and Digestive enzymes, Table S12: Metabolic detoxification, Table S13: Circadian Genes, Table S14: Behavioural Genes, Table S15: Reproduction related genes, Table S16: Insecticidal targets, Table S17: Immune related genes, Table S18: Insect effectors, Table S19: RNAi Machinery, Table S20: Primers used for the study.

## References

[1] Foley J.A., Ramankutty N., Brauman K.A., Cassidy E.S., Gerber J.S., Johnston M., Mueller N.D., O’Connell C., Ray D.K., West P.C. (2011). Solutions for a cultivated planet. Nature.

[2] Godfray H.C.J., Beddington J.R., Crute I.R., Haddad L., Lawrence D., Muir J.F., Pretty J., Robinson S., Thomas S.M., Toulmin C. (2010). Food security: The challenge of feeding 9 billion people. Science.

[3] FAO (2016). Maize, Rice, Wheat: A Guide to Sustainable Cereal Production.

[4] FAOSTAT Food and Agriculture Organization of the United Nations Statistics Division. http://www.fao.org/faostat/en/#home.

[5] Pathak M.D., Khan Z.R. (1994). Insect Pests of Rice.

[6] Rubia E.G., Shepard B.M., Yambao E.B., Ingram K.T., Arida G.S., de Penning V. (1989). Stem borer damage and grain yield of flooded rice. J. Plant Prot. Trop..

[7] Shepard B.M. (1995). Rice-Feeding Insects of Tropical Asia.

[8] Taylor B. (1988). The impact of yellow stem-borer, *Scirpophaga incertulas* (Walker) (Lepidoptera: Pyralidae), on deepwater rice, with special reference to Bangladesh. Bull. Entomol. Res..

[9] Taylor B. (1996). *Scirpophaga incertulas* (Walker) (Lepidoptera: Pyralidae) and deepwater rice—An integrated view. Crop Prot..

[10] Datta S.K., A Promising Debut for Bt Hybrid Rice (2000). Insect Resistance. Inf. Syst. Biotechnol. News Rep. Sept..

[11] Evans J.D., Gundersen-Rindal D. (2003). Beenomes to *Bombyx*: Future directions in applied insect genomics. Genome Biol..

[12] Li F., Zhao X., Li M., He K., Huang C., Zhou Y., Li Z., Walters J.R. (2019). Insect genomes: Progress and challenges. Insect Mol. Biol..

[13] Renuka P., Madhav M.S., Padmakumari A.P., Barbadikar K.M., Mangrauthia S.K., Sudhakara Rao K.V., Marla S.S., Babu V.R. (2017). RNA-seq of rice yellow stem borer *Scirpophaga incertulas* reveals molecular insights during four larval developmental stages. G3 Genes Genomes Genet..

[14] Padmakumari A.P., Katti G., Sailaja V., Padmavathi C., Lakshmi V.J., Prabhakar M., Prasad Y.G. (2013). Delineation of larval instars in field populations of rice yellow stem borer, *Scirpophaga incertulas* (Walk.). ORYZA Int. J. Rice.

[15] Thiel T., Michalek W., Varshney R.K., Graner A. (2003). Exploiting EST databases for the development and characterization of gene-derived SSR-markers in barley (*Hordeum vulgare* L.). Theor. Appl. Genet..

[16] Zala H.N., Kulkarni K.S., Bosamia T.C., Shukla Y.M., Kumar S., Fougat R.S., Patel A. (2017). Development and validation of EST derived SSR markers with relevance to downy mildew (*Sclerospora graminicola* Sacc.) resistance in pearl millet [*Pennisetum glaucum* (L.) R. Br.]. J. Plant Biochem. Biotechnol..

[17] Kumar S., Stecher G., Tamura K. (2016). MEGA7: Molecular Evolutionary Genetics Analysis Version 7.0 for Bigger Datasets. Mol. Biol. Evol..

[18] Galbraith D.W., Harkins K.R., Maddox J.M., Ayres N.M., Sharma D.P., Firoozabady E. (1983). Rapid flow cytometric analysis of the cell cycle in intact plant tissues. Science.

[19] Lauressergues D., Couzigou J.M., San Clemente H., Martinez Y., Dunand C., Bécard G., Combier J.P. (2015). Primary transcripts of microRNAs encode regulatory peptides. Nature.

[20] Mirzoyan Z., Pandur P. (2013). The Iroquois Complex Is Required in the Dorsal Mesoderm to Ensure Normal Heart Development in Drosophila. PLoS ONE.

[21] Ashok M., Turner C., Wilson T.G. (1998). Insect juvenile hormone resistance gene homology with the bHLH-PAS family of transcriptional regulators. Proc. Natl. Acad. Sci. USA.

[22] Jindra M., Palli S.R., Riddiford L.M. (2013). The juvenile hormone signaling pathway in insect development. Annu. Rev. Entomol..

[23] Ma L., Zhang W., Liu C., Chen L., Xu Y., Xiao H., Liang G. (2018). Methoprene-tolerant (Met) is indispensable for larval metamorphosis and female reproduction in the cotton bollworm helicoverpa armigera. Front. Physiol..

[24] Lin X., Yao Y., Wang B. (2015). Methoprene-tolerant (Met) and Krüpple-homologue 1 (Kr-h1) are required for ovariole development and egg maturation in the brown plant hopper. Sci. Rep..

[25] Muramatsu D., Kinjoh T., Shinoda T., Hiruma K. (2008). The role of 20-hydroxyecdysone and juvenile hormone in pupal commitment of the epidermis of the silkworm, Bombyx mori. Mech. Dev..

[26] Cheng D., Qian W., Meng M., Wang Y., Peng J., Xia Q. (2014). Identification and expression profiling of the btb domain-containing protein gene family in the Silkworm, Bombyx mori. Int. J. Genomics.

[27] Matsumoto A., Ukai-Tadenuma M., Yamada R.G., Houl J., Uno K.D., Kasukawa T., Dauwalder B., Itoh T.Q., Takahashi K., Ueda R. (2007). A functional genomics strategy reveals clockwork orange as a transcriptional regulator in the Drosophila circadian clock. Genes Dev..

[28] Tufail M., Takeda M. (2008). Molecular characteristics of insect vitellogenins. J. Insect Physiol..

[29] Lu K., Wang Y., Chen X., Zhang X., Li W., Cheng Y., Li Y., Zhou J., You K., Song Y. (2019). Adipokinetic Hormone Receptor Mediates Trehalose Homeostasis to Promote Vitellogenin Uptake by Oocytes in Nilaparvata lugens. Front. Physiol..

[30] Terakita A. (2005). The opsins. Genome Biol..

[31] Bockaert J., Pin J.P. (1999). Molecular tinkering of G protein-coupled receptors: An evolutionary success. EMBO J..

[32] Xu Y.L., He P., Zhang L., Fang S.Q., Dong S.L., Zhang Y.J., Li F. (2009). Large-scale identification of odorant-binding proteins and chemosensory proteins from expressed sequence tags in insects. BMC Genom..

[33] Vieira F.G., Rozas J. (2011). Comparative genomics of the odorant-binding and chemosensory protein gene families across the arthropoda: Origin and evolutionary history of the chemosensory system. Genome Biol. Evol..

[34] Silbering A.F., Benton R. (2010). Ionotropic and metabotropic mechanisms in chemoreception: Chance or design?. EMBO Rep..

[35] Zou Z., Lopez D.L., Kanost M.R., Evans J.D., Jiang H. (2006). Comparative analysis of serine protease-related genes in the honey bee genome: Possible involvement in embryonic development and innate immunity. Insect Mol. Biol..

[36] Lin H., Xia X., Yu L., Vasseur L., Gurr G.M., Yao F., Yang G., You M. (2015). Genome-wide identification and expression profiling of serine proteases and homologs in the diamondback moth, *Plutella xylostella* (L.). BMC Genom..

[37] Ffrench-Constant R.H., Daborn P.J., Le Goff G. (2004). The genetics and genomics of insecticide resistance. Trends Genet..

[38] Ono H., Rewitz K.F., Shinoda T., Itoyama K., Petryk A., Rybczynski R., Jarcho M., Warren J.T., Marqués G., Shimell M.J. (2006). Spook and Spookier code for stage-specific components of the ecdysone biosynthetic pathway in Diptera. Dev. Biol..

[39] Després L., David J.P., Gallet C. (2007). The evolutionary ecology of insect resistance to plant chemicals. Trends Ecol. Evol..

[40] Zhou W.W., Liang Q.M., Xu Y., Gurr G.M., Bao Y.Y., Zhou X.P., Zhang C.X., Cheng J., Zhu Z.R. (2013). Genomic Insights into the Glutathione S-Transferase Gene Family of Two Rice Planthoppers, *Nilaparvata lugens* (Stål) and *Sogatella furcifera* (Horváth) (Hemiptera: Delphacidae). PLoS ONE.

[41] Teixeira L., Duggan B., Senn R. (2016). Lepidoptera Working Group Session 2B Welcome Lepidoptera WG Members!. Representative.

[42] Nicholson S.J., Nickerson M.L., Dean M., Song Y., Hoyt P.R., Rhee H., Kim C., Puterka G.J. (2015). The genome of *Diuraphis noxia*, a global aphid pest of small grains. BMC Genom..

[43] Zhu J., Jiang F., Wang X., Yang P., Bao Y., Zhao W., Wang W., Lu H., Wang Q., Cui N. (2017). Genome sequence of the small brown planthopper, *Laodelphax striatellus*. Gigascience.

[44] Saunders D.S. (2002). Insect Clocks.

[45] Smith L.A., Peixoto A.A., Kramer E.M., Villella A., Hall J.C. (1998). Courtship and visual defects of cacophony mutants reveal functional complexity of a calcium-channel α1 subunit in Drosophila. Genetics.

[46] Hall J.C. (1994). The mating of a fly. Science.

[47] Peixoto A.A., Hall J.C. (1998). Analysis of temperature-sensitive mutants reveals new genes involved in the courtship song of Drosophila. Genetics.

[48] Roshchina V.V. (2016). New trends and perspectives in the evolution of neurotransmitters in microbial, plant, and animal cells. Advances in Experimental Medicine and Biology.

[49] Kingsolver M.B., Hardy R.W. (2012). Making connections in insect innate immunity. Proc. Natl. Acad. Sci. USA.

[50] Yoshida H., Kinoshita K., Ashida M. (1996). Purification of a peptidoglycan recognition protein from hemolymph of the silkworm, *Bombyx mori*. J. Biol. Chem..

[51] Ribeiro J.M.C., Sarkis J.J.F., Rossignol P.A., Spielman A. (1984). Salivary apyrase of *Aedes Aegypti*: Characterization and secretory fate. Comp. Biochem. Physiol. Part B Biochem..

[52] Ribeiro J.M.C. (2000). Blood-feeding in mosquitoes: Probing time and salivary gland anti-haemostatic activities in representatives of three genera (*Aedes*, *Anopheles*, *Culex*). Med. Vet. Entomol..

[53] Nazzi F., Brown S.P., Annoscia D., Del Piccolo F., Di Prisco G., Varricchio P., Della Vedova G., Cattonaro F., Caprio E., Pennacchio F. (2012). Synergistic parasite-pathogen interactions mediated by host immunity can drive the collapse of honeybee colonies. PLoS Pathog..

[54] Ferreira Á.G., Naylor H., Esteves S.S., Pais I.S., Martins N.E., Teixeira L. (2014). The Toll-Dorsal Pathway Is Required for Resistance to Viral Oral Infection in *Drosophila*. PLoS Pathog..

[55] Xi Z., Ramirez J.L., Dimopoulos G. (2008). The *Aedes aegypti* toll pathway controls dengue virus infection. PLoS Pathog..

[56] War A.R., Paulraj M.G., Ahmad T., Buhroo A.A., Hussain B., Ignacimuthu S., Sharma H.C. (2012). Mechanisms of plant defense against insect herbivores. Plant Signal. Behav..

[57] Howe G.A., Jander G. (2008). Plant immunity to insect herbivores. Annu. Rev. Plant Biol..

[58] Wu J., Baldwin I.T. (2010). New insights into plant responses to the attack from insect herbivores. Annu. Rev. Genet..

[59] Ghildiyal M., Zamore P.D. (2009). Small silencing RNAs: An expanding universe. Nat. Rev. Genet..

[60] Siomi M.C., Sato K., Pezic D., Aravin A.A. (2011). PIWI-interacting small RNAs: The vanguard of genome defence. Nat. Rev. Mol. Cell Biol..

[61] Ding S., Wang S., He K., Jiang M., Li F. (2017). Large-scale analysis reveals that the genome features of simple sequence repeats are generally conserved at the family level in insects. BMC Genomics.

[62] Ohbayashi F., Shimada T., Sugasaki T., Kawai S., Mita K., Oshiki T., Abe H. (1998). Molecular structure of the copia-like retrotransposable element Yokozuna on the W chromosome of the silkworm, *Bombyx mori*. Genes Genet. Syst..

[63] Oakeshott J.G., Claudianos C., Russell R.J., Robin G.C. (1999). Carboxyl/cholinesterases: A case study of the evolution of a successful multigene family. BioEssays.

[64] Zhan S., Merlin C., Boore J.L., Reppert S.M. (2011). The monarch butterfly genome yields insights into long-distance migration. Cell.

[65] Dasmahapatra K.K., Walters J.R., Briscoe A.D., Davey J.W., Whibley A., Nadeau N.J., Zimin A.V., Salazar C., Ferguson L.C., Martin S.H. (2012). Butterfly genome reveals promiscuous exchange of mimicry adaptations among species. Nature.

[66] Nishikawa H., Iijima T., Kajitani R., Yamaguchi J., Ando T., Suzuki Y., Sugano S., Fujiyama A., Kosugi S., Hirakawa H. (2015). A genetic mechanism for female-limited Batesian mimicry in *Papilio* butterfly. Nat. Genet..

[67] Yin C., Liu Y., Liu J., Xiao H., Huang S., Lin Y., Han Z., Li F. (2014). ChiloDB: A genomic and transcriptome database for an important rice insect pest *Chilo suppressalis*. Database.

[68] Gouin A., Bretaudeau A., Nam K., Gimenez S., Aury J.M., Duvic B., Hilliou F., Durand N., Montagné N., Darboux I. (2017). Two genomes of highly polyphagous lepidopteran pests (*Spodoptera frugiperda*, Noctuidae) with different host-plant ranges. Sci. Rep..

[69] Bao Y.Y., Qu L.Y., Zhao D., Chen L.B., Jin H.Y., Xu L.M., Cheng J.A., Zhang C.X. (2013). The genome- and transcriptome-wide analysis of innate immunity in the brown planthopper, *Nilaparvata lugens*. BMC Genom..

[70] Xue J., Zhou X., Zhang C.X., Yu L.L., Fan H.W., Wang Z., Xu H.J., Xi Y., Zhu Z.R., Zhou W.W. (2014). Genomes of the rice pest brown planthopper and its endosymbionts reveal complex complementary contributions for host adaptation. Genome Biol..

[71] Kakumani P.K., Malhotra P., Mukherjee S.K., Bhatnagar R.K. (2014). A draft genome assembly of the army worm, *Spodoptera frugiperda*. Genom..

[72] Keightley P.D., Trivedi U., Thomson M., Oliver F., Kumar S., Blaxter M.L. (2009). Analysis of the genome sequences of three *Drosophila melanogaster* spontaneous mutation accumulation lines. Genome Res..

[73] Wang L., Tang N., Gao X., Chang Z., Zhang L., Zhou G., Guo D., Zeng Z., Li W., Akinyemi I.A. (2017). Genome sequence of a rice pest, the white-backed planthopper (*Sogatella furcifera*). Gigascience.

[74] Kelley J.L., Peyton J.T., Fiston-Lavier A.S., Teets N.M., Yee M.C., Johnston J.S., Bustamante C.D., Lee R.E., Denlinger D.L. (2014). Compact genome of the Antarctic midge is likely an adaptation to an extreme environment. Nat. Commun..

[75] Wang X., Fang X., Yang P., Jiang X., Jiang F., Zhao D., Li B., Cui F., Wei J., Ma C. (2014). The locust genome provides insight into swarm formation and long-distance flight. Nat. Commun..

[76] Richards S., Gibbs R.A., Weinstock G.M., Brown S., Denell R., Beeman R.W., Gibbs R., Bucher G., Friedrich M., Grimmelikhuijzen C.J.P. (2008). The genome of the model beetle and pest *Tribolium castaneum*. Nature.

[77] Osanai-Futahashi M., Suetsugu Y., Mita K., Fujiwara H. (2008). Genome-wide screening and characterization of transposable elements and their distribution analysis in the silkworm. Bombyx mori. Insect Biochem. Mol. Biol..

[78] Huang L., Cheng T., Xu P., Fang T., Xia Q. (2012). *Bombyx mori* transcription factors: Genome-wide identification, expression profiles and response to pathogens by microarray analysis. J. Insect Sci..

[79] Wegner M., Stolt C.C. (2005). From stem cells to neurons and glia: A Soxist’s view of neural development. Trends Neurosci..

[80] Laudet V., Stehelin D., Clevers H. (1993). Ancestry and diversity of the HMG box superfamily. Nucleic Acids Res..

[81] Kiefer J.C. (2007). Back to basics: Sox genes. Dev. Dyn..

[82] McKimmie C., Woerfel G., Russell S. (2005). Conserved genomic organisation of Group B Sox genes in insects. BMC Genet..

[83] Crémazy F., Berta P., Girard F. (2001). Genome-wide analysis of Sox genes in *Drosophila melanogaster*. Mech. Dev..

[84] Wilson M.J., Dearden P.K. (2008). Evolution of the insect Sox genes. BMC Evol. Biol..

[85] Lu K., Shu Y., Zhou J., Zhang X., Zhang X., Chen M., Yao Q., Zhou Q., Zhang W. (2015). Molecular characterization and RNA interference analysis of vitellogenin receptor from *Nilaparvata lugens* (Stål). J. Insect Physiol..

[86] Hu K., Tian P., Tang Y., Yang L., Qiu L., He H., Ding W., Li Z., Li Y. (2019). Molecular Characterization of Vitellogenin and Its Receptor in *Sogatella furcifera*, and Their Function in Oocyte Maturation. Front. Physiol..

[87] Zhang J., Xing Y., Li Y., Yin C., Ge C., Li F. (2015). DNA methyltransferases have an essential role in female fecundity in brown planthopper, *Nilaparvata lugens*. Biochem. Biophys. Res. Commun..

[88] Zhang M., Chen J.L., Liang S.K., Li G.H., Wang F.H., Ahmad I. (2015). Differentially methylated genomic fragments related with sexual dimorphism of rice pests, *Sogatella furcifera*. Insect Sci..

[89] Futahashi R., Kawahara-Miki R., Kinoshita M., Yoshitake K., Yajima S., Arikawa K., Fukatsu T. (2015). Extraordinary diversity of visual opsin genes in dragonflies. Proc. Natl. Acad. Sci. USA.

[90] Briscoe A.D. (2008). Reconstructing the ancestral butterfly eye: Focus on the opsins. J. Exp. Biol..

[91] Zhu H., Yuan Q., Froy O., Casselman A., Reppert S.M. (2005). The two CRYs of the butterfly. Curr. Biol..

[92] Yan S., Ni H., Li H., Zhang J., Liu X., Zhang Q. (2013). Molecular cloning, characterization, and mRNA expression of two Cryptochrome genes in *Helicoverpa armigera* (Lepidoptera: Noctuidae). J. Econ. Entomol..

[93] Yuan Q., Metterville D., Briscoe A.D., Reppert S.M. (2007). Insect cryptochromes: Gene duplication and loss define diverse ways to construct insect circadian clocks. Mol. Biol. Evol..

[94] Rubin E.B., Shemesh Y., Cohen M., Elgavish S., Robertson H.M., Bloch G. (2006). Molecular and phylogenetic analyses reveal mammalian-like clockwork in the honey bee (*Apis mellifera*) and shed new light on the molecular evolution of the circadian clock. Genome Res..

[95] Fujikawa K., Seno K., Ozaki M. (2006). A novel takeout-like protein expressed in the taste and olfactory organs of the blowfly, *Phormia regina*. FEBS J..

[96] Du J., Hiruma K., Riddiford L.M. (2003). A novel gene in the takeout gene family is regulated by hormones and nutrients in *Manduca* larval epidermis. Insect Biochem. Mol. Biol..

[97] Smadja C., Shi P., Butlin R.K., Robertson H.M. (2009). Large gene family expansions and adaptive evolution for odorant and gustatory receptors in the pea aphid, *Acyrthosiphon pisum*. Mol. Biol. Evol..

[98] Terrapon N., Li C., Robertson H.M., Ji L., Meng X., Booth W., Chen Z., Childers C.P., Glastad K.M., Gokhale K. (2014). Molecular traces of alternative social organization in a termite genome. Nat. Commun..

[99] Croset V., Rytz R., Cummins S.F., Budd A., Brawand D., Kaessmann H., Gibson T.J., Benton R. (2010). Ancient protostome origin of chemosensory ionotropic glutamate receptors and the evolution of insect taste and olfaction. PLoS Genet..

[100] Gong D.P., Zhang H.J., Zhao P., Xia Q.Y., Xiang Z.H. (2009). The odorant binding protein gene family from the genome of silkworm, *bombyx mori*. BMC Genom..

[101] Xu W., Papanicolaou A., Liu N.Y., Dong S.L., Anderson A. (2015). Chemosensory receptor genes in the Oriental tobacco budworm *Helicoverpa assulta*. Insect Mol. Biol..

[102] Vieira F.G., Sánchez-Gracia A., Rozas J. (2007). Comparative genomic analysis of the odorant-binding protein family in 12 *Drosophila* genomes: Purifying selection and birth-and-death evolution. Genome Biol..

[103] Forêt S., Maleszka R. (2006). Function and evolution of a gene family encoding odorant binding-like proteins in a social insect, the honey bee (*Apis mellifera*). Genome Res..

[104] Chen D., Qu C., Hewes R.S. (2014). Neuronal remodeling during metamorphosis is regulated by the alan shepard (shep) gene in *Drosophila melanogaster*. Genetics.

[105] Ma E., Xu T., Haddada G.G. (1999). Gene regulation by O2 deprivation: An anoxia-regulated novel gene in *Drosophila melanogaster*. Mol. Brain Res..

[106] Olesnicky E.C., Antonacci S., Popitsch N., Lybecker M.C., Titus M.B., Valadez R., Derkach P.G., Marean A., Miller K., Mathai S.K. (2018). Shep interacts with posttranscriptional regulators to control dendrite morphogenesis in sensory neurons. Dev. Biol..

[107] Majeed Z.R., Abdeljaber E., Soveland R., Cornwell K., Bankemper A., Koch F., Cooper R.L. (2016). Modulatory Action by the Serotonergic System: Behavior and Neurophysiology in *Drosophila melanogaster*. Neural Plast..

[108] Paulk A.C., Dacks A.M., Phillips-Portillo J., Fellous J.M., Gronenberg W. (2009). Visual processing in the central bee brain. J. Neurosci..

[109] Dacks A.M., Christensen T.A., Hildebrand J.G. (2008). Modulation of olfactory information processing in the antennal lobe of *Manduca sexta* by serotonin. J. Neurophysiol..

[110] Andrés M., Seifert M., Spalthoff C., Warren B., Weiss L., Giraldo D., Winkler M., Pauls S., Göpfert M.C. (2016). Auditory Efferent System Modulates Mosquito Hearing. Curr. Biol..

[111] French A.S., Simcock K.L., Rolke D., Gartside S.E., Blenau W., Wright G.A. (2014). The role of serotonin in feeding and gut contractions in the honeybee. J. Insect Physiol..

[112] Giese M., Gestrich J., Massah A., Peterle J., Wei H.Y., Stengl M. (2018). GABA- and serotonin-expressing neurons take part in inhibitory as well as excitatory input pathways to the circadian clock of the Madeira cockroach *Rhyparobia maderae*. Eur. J. Neurosci..

[113] Sitaraman D., Zars M., LaFerriere H., Chen Y.C., Sable-Smith A., Kitamoto T., Rottinghaus G.E., Zars T. (2008). Serotonin is necessary for place memory in *Drosophila*. Proc. Natl. Acad. Sci. USA.

[114] Sitaraman D., LaFerriere H., Birman S., Zars T. (2012). Serotonin is critical for rewarded olfactory short-term memory in Drosophila. J. Neurogenet..

[115] Raymond-Delpech V., Matsuda K., Sattelle B.M., Rauh J.J., Sattelle D.B. (2005). Ion channels: Molecular targets of neuroactive insecticides. Invertebr. Neurosci..

[116] Jones A.K., Bera A.N., Lees K., Sattelle D.B. (2010). The cys-loop ligand-gated ion channel gene superfamily of the parasitoid wasp, *Nasonia vitripennis*. Heredity.

[117] Lemaitre B., Hoffmann J. (2007). The host defense of *Drosophila melanogaster*. Annu. Rev. Immunol..

[118] Ashida M., Brey P.T. (1998). Recent advances in research on the insect prophenoloxidase cascade. Molecular Mechanisms of Immune Responses in Insects.

[119] Wu K., Zhang J., Zhang Q., Zhu S., Shao Q., Clark K.D., Liu Y., Ling E. (2015). Plant phenolics are detoxified by prophenoloxidase in the insect gut. Sci. Rep..

[120] Hultmark D., Engström A., Andersson K., Steiner H., Bennich H., Boman H.G. (1983). Insect immunity. Attacins, a family of antibacterial proteins from *Hyalophora cecropia*. EMBO J..

[121] Cheng T., Zhao P., Liu C., Xu P., Gao Z., Xia Q., Xiang Z. (2006). Structures, regulatory regions, and inductive expression patterns of antimicrobial peptide genes in the silkworm *Bombyx mori*. Genomics.

[122] Zou Z., Evans J.D., Lu Z., Zhao P., Williams M., Sumathipala N., Hetru C., Hultmark D., Jiang H. (2007). Comparative genomic analysis of the Tribolium immune system. Genome Biol..

[123] Wu S., Peiffer M., Luthe D.S., Felton G.W. (2012). ATP hydrolyzing salivary enzymes of caterpillars suppress plant defenses. PLoS ONE.

[124] Funk C.J. (2001). Alkaline phosphatase activity in whitefly salivary glands and saliva. Arch. Insect Biochem. Physiol..

[125] Mattiacci L., Dicke M., Posthumus M.A. (1995). β-Glucosidase: An elicitor of herbivore-induced plant odor that attracts host-searching parasitic wasps. Proc. Natl. Acad. Sci. USA.

[126] Chen C.Y., Liu Y.Q., Song W.M., Chen D.Y., Chen F.Y., Chen X.Y., Chen Z.W., Ge S.X., Wang C.Z., Zhan S. (2019). An effector from cotton bollworm oral secretion impairs host plant defense signaling. Proc. Natl. Acad. Sci. USA.

[127] Kola V.S.R., Pichili R., Padmakumari A.P., Mangrauthia S.K., Balachandran S.M., Madhav M.S. (2019). Knockdown of acetylcholinesterase (AChE) gene in rice yellow stem borer, *Scirpophaga incertulas* (Walker) through RNA interference. Agric. Gene.

[128] Diehl S.R., Bush G.L. (1984). An evolutionary and applied perspective of insect biotypes. Annu. Rev. Entomol..

[129] Ratnieks F.L.W., Keller L. (1998). Queen control of egg fertilization in the honey bee. Behav. Ecol. Sociobiol..

[130] Abe J., Kamimura Y., Shimada M., West S.A. (2009). Extremely female-biased primary sex ratio and precisely constant male production in a parasitoid wasp *Melittobia*. Anim. Behav..

[131] Khidr S.K., Hardy I.C.W., Zaviezo T., Mayes S. (2014). Development of microsatellite markers and detection of genetic variation between *Goniozus* wasp populations. J. Insect Sci..

