# Supplementary material for: The Draft Genome of Yellow Stem Borer, an Agriculturally Important Pest, Provides Molecular Insights into Its Biology, Development and Specificity Towards Rice for Infestation
Source: Insects. 2021 Jun 19;12(6):563. doi: 10.3390/insects12060563 (PMC8234988; doi:10.3390/insects12060563)
Supplement: Supplementary file 1 [file insects-12-00563-s001.zip › YSB_Supplementary Figures.pdf]

# Draft genome of Yellow Stem borer, an agriculturally important pest provides molecular insights into its biology, development and specificity towards rice for infestation

Divya Kattupalli[1#], Kalyani M. Barbadikar[1#], Vishalakshi B[1], Suneel B[1], Athulya R[2], Padmakumari A.P[2], Swati Saxena[3], Kishor Gaikwad[3], Sridhar Y [2], Premalatha K[4], Sheshu Madhav Maganti[1]*

## Supplementary Figures

### Figure 1: Gene Ontology Analysis

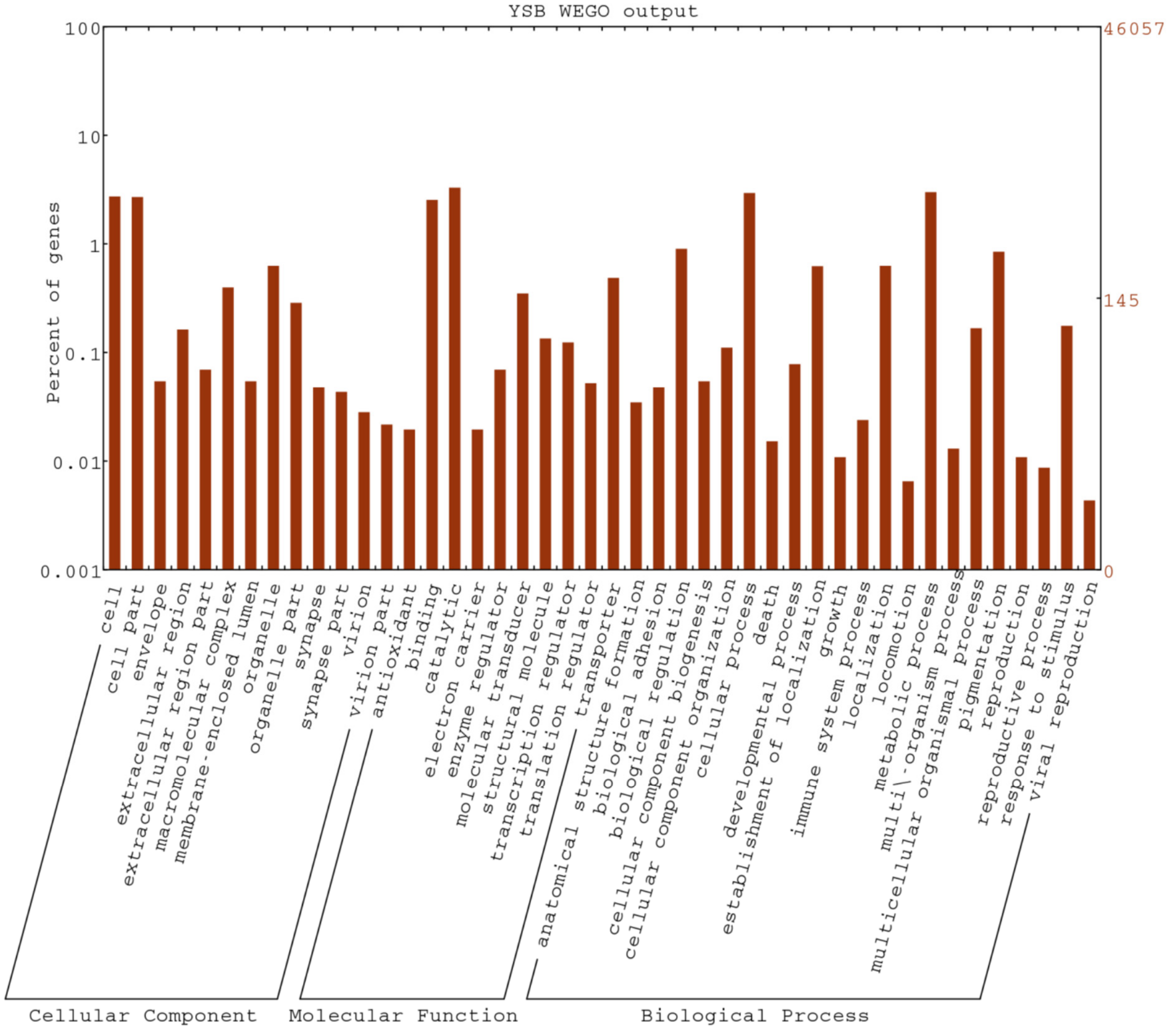

**Figure 2:** COG class distribution for YSB sample

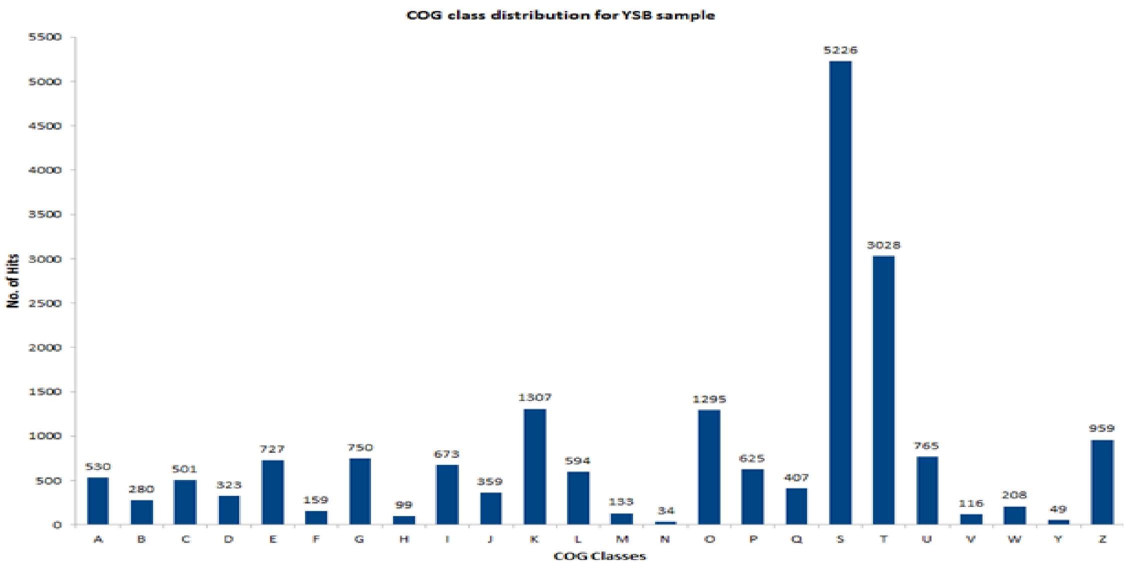


**Supplementary Figure 3:** Phylogenetic relationships of the Ecdysone receptor gene in *Scirpophaga incertulas* compared to other insects.

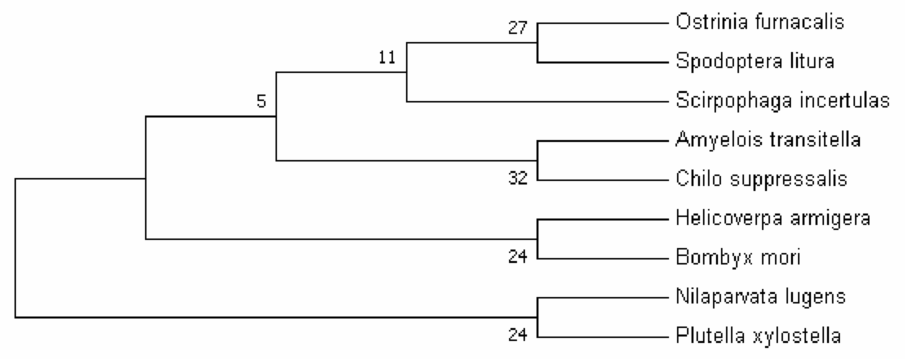


**Supplementary Figure 4:** Phylogenetic relationships of the Pheromone binding gene in *Scirpophaga incertulas* compared to other insects.

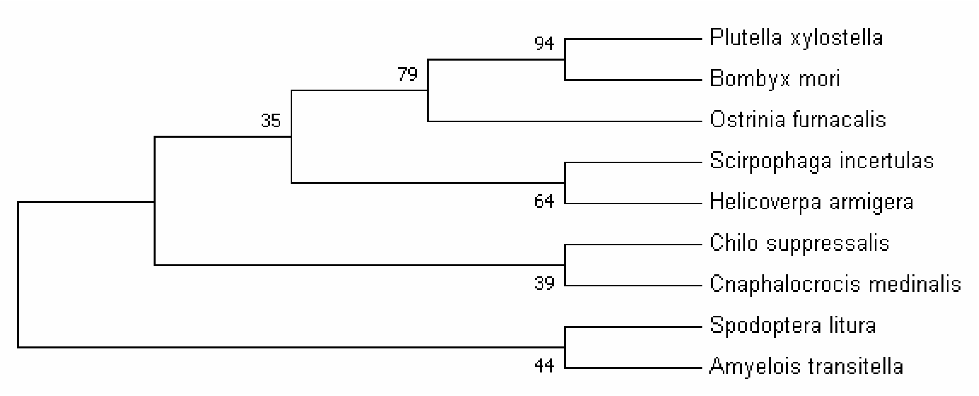


**Supplementary Figure 5:** Phylogenetic relationships of the Vitellogenin gene in *Scirpophaga incertulas* compared to other insects.

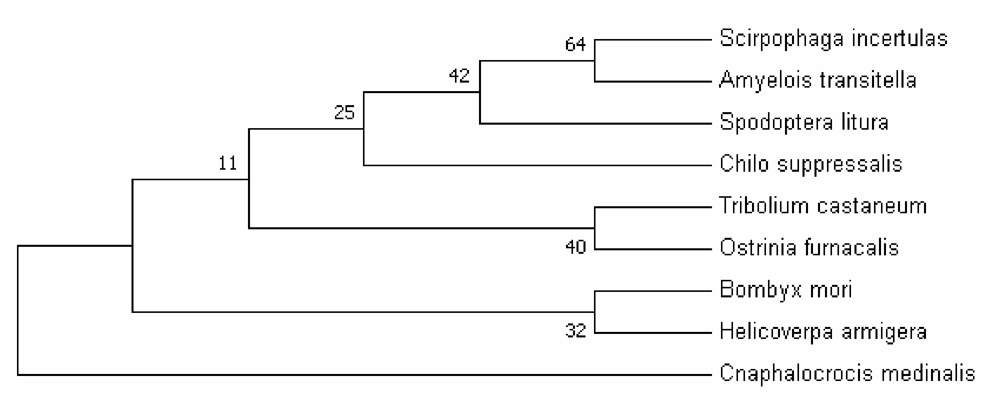


**Supplementary Figure 6:** Phylogenetic relationships of the Long wavelength opsin gene in *Scirpophaga incertulas* compared to other insects.

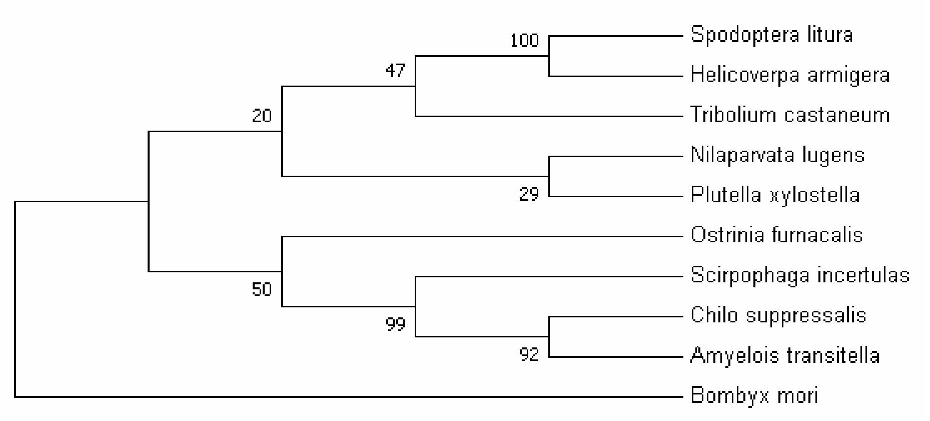


**Supplementary Figure 7:** Phylogenetic relationships of the Metabotropic chemosensory receptor gene in *Scirpophaga incertulas* compared to other insects.

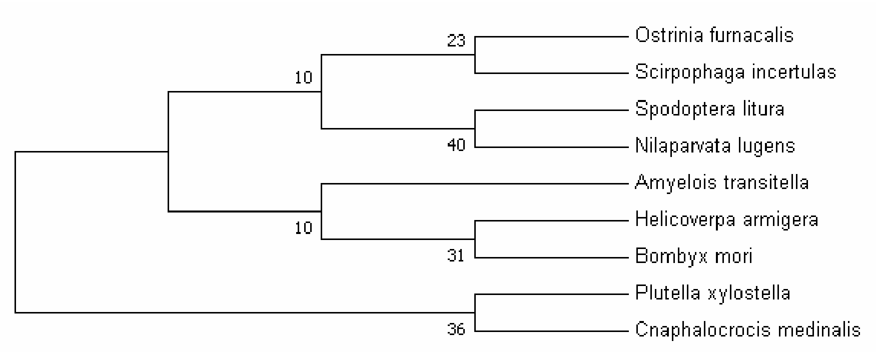


**Supplementary Figure 8:** Phylogenetic relationships of the Aminopeptidase N gene in *Scirpophaga incertulas* compared to other insects.

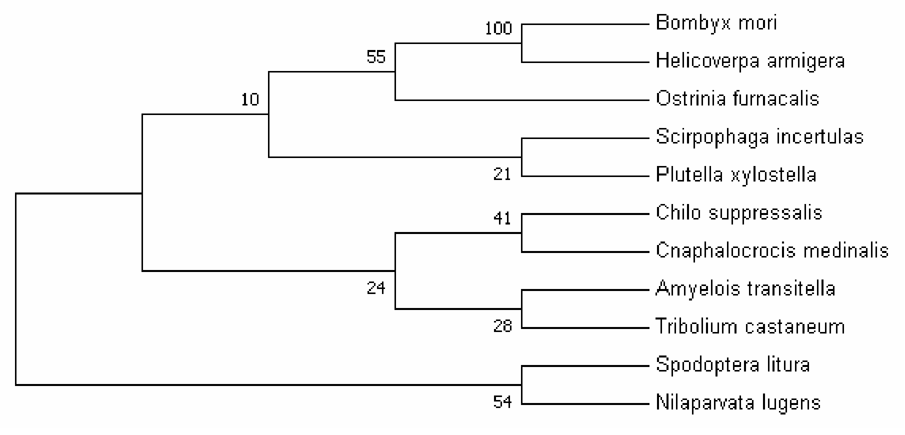


**Supplementary Figure 9:** Phylogenetic relationships of the Serine protease gene in *Scirpophaga incertulas* compared to other insects.

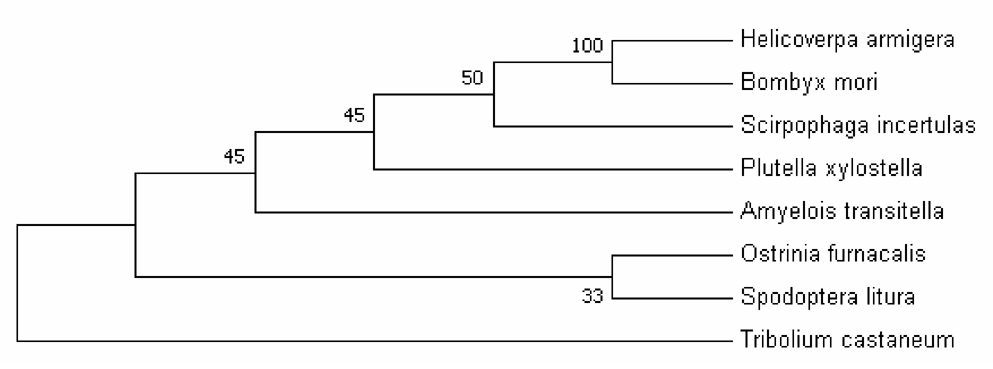


**Supplementary Figure 10:** Phylogenetic relationships of the Alpha-Amylase gene in *Scirpophaga incertulas* compared to other insects.

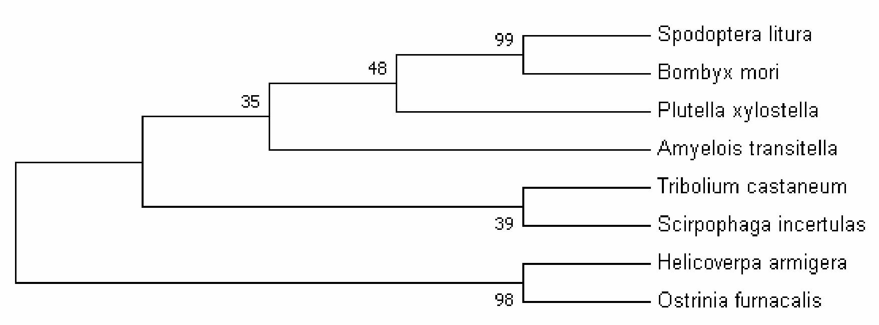


**Supplementary Figure 11:** Phylogenetic relationships of the Cacophony gene in *Scirpophaga incertulas* compared to other insects.

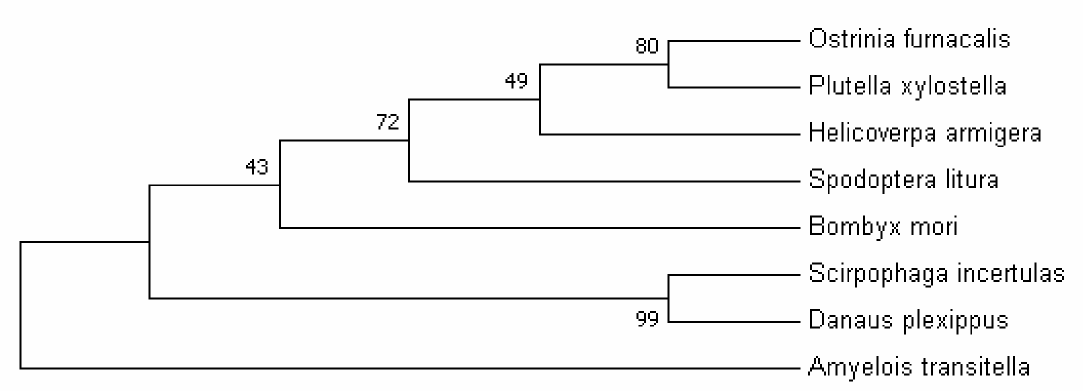

**Supplementary Figure 12:** Phylogenetic relationships of the Alan Shepard gene in *Scirpophaga incertulas* compared to other insects.

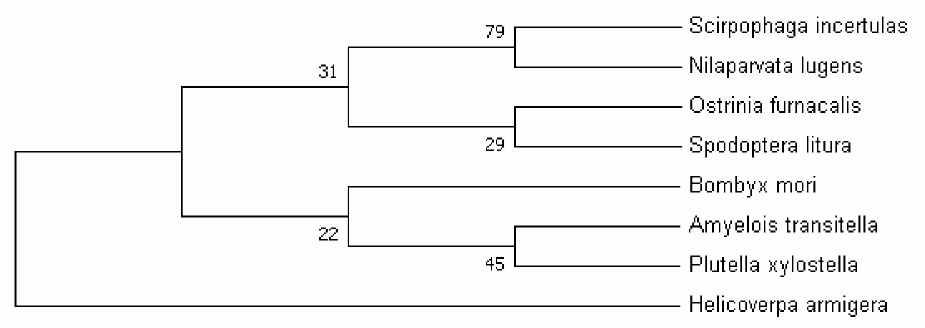


**Supplementary Figure 13:** Phylogenetic relationships of the Annoxia gene in *Scirpophaga incertulas* compared to other insects.

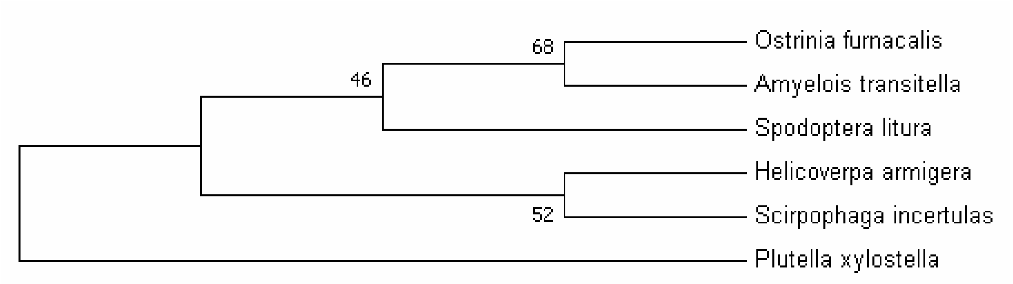


**Supplementary Figure 14:** Phylogenetic relationships of the Serotonin receptor gene in *Scirpophaga incertulas* compared to other insects.

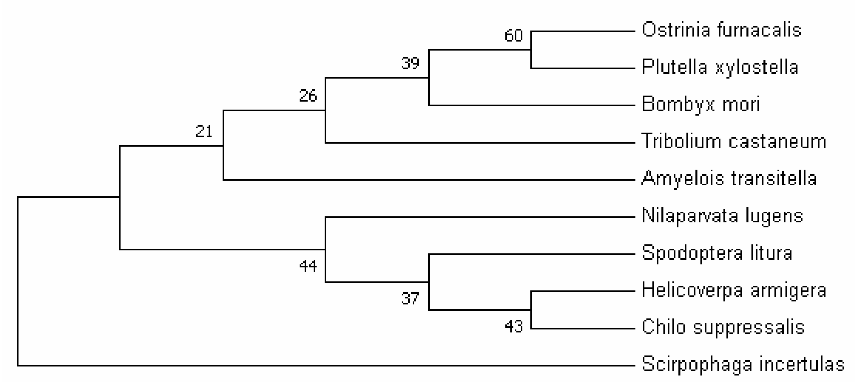

**Supplementary Figure 15:** Phylogenetic relationships of the Gamma-aminobutyric acid (GABA) gene in *Scirpophaga incertulas* compared to other insects.

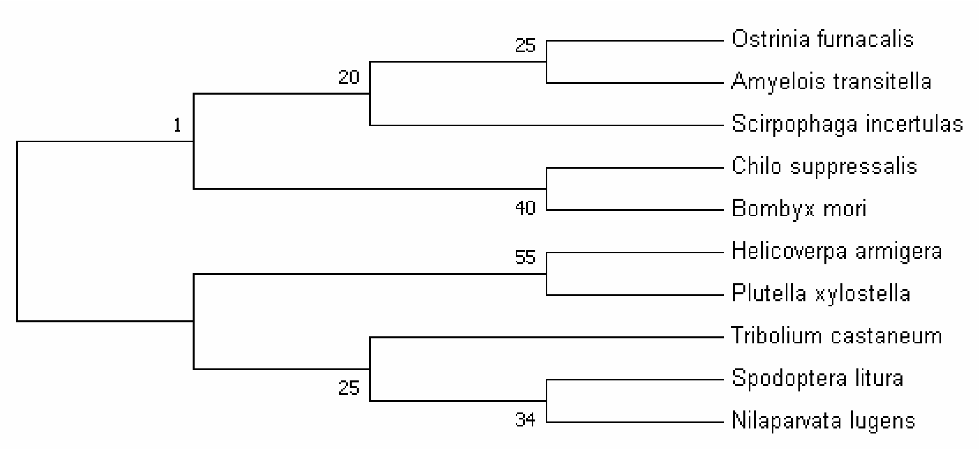


**Supplementary Figure 16:** Phylogenetic relationships of the Venom gene in *Scirpophaga incertulas* compared to other insects.

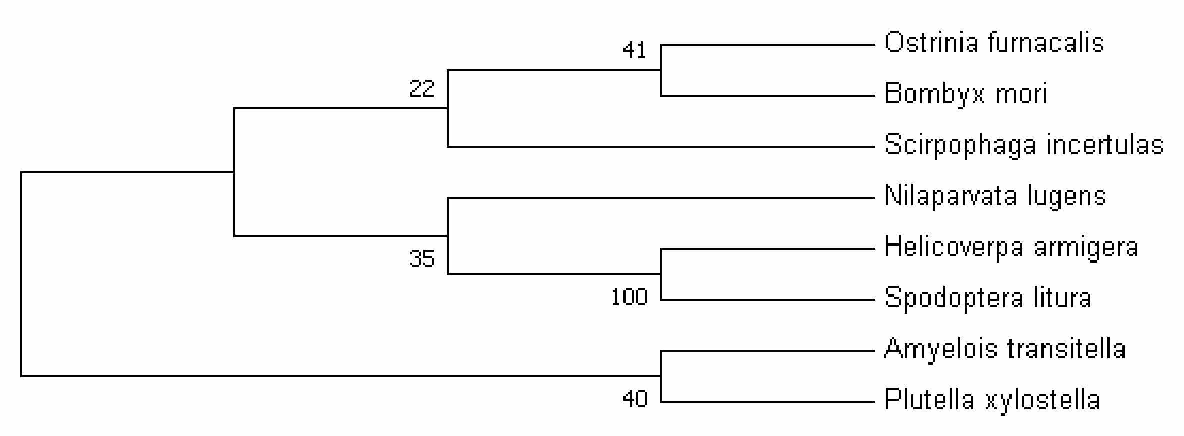


**Supplementary Figure 17: Evolutionary timeline analysis**

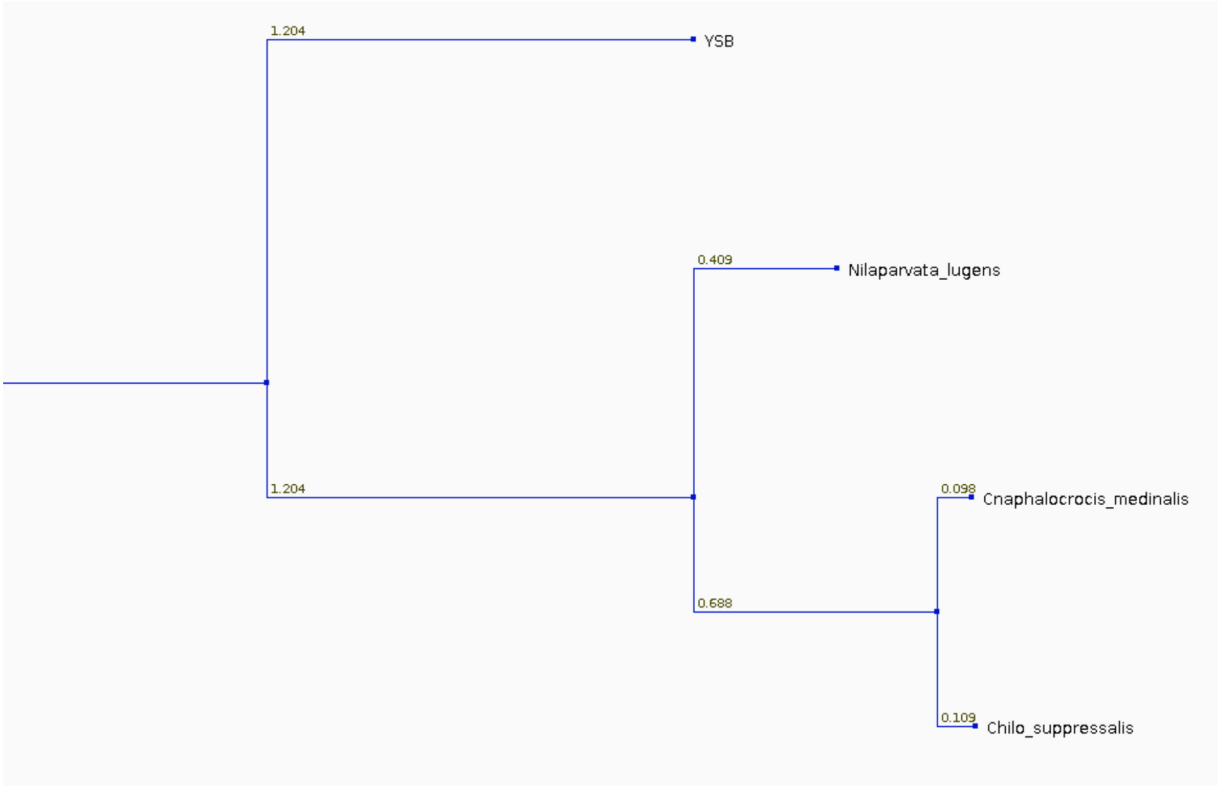
1.204
YSB
0.409
Nilaparvata_lugens
1.204
0.098
Cnaphalocrocis_medinalis
0.688
0.109
Chilo_suppressalis
